# The concept of intersectionality in bioethics: a systematic review

**DOI:** 10.1186/s12910-024-01057-5

**Published:** 2024-05-23

**Authors:** Lisa Brünig, Hannes Kahrass, Sabine Salloch

**Affiliations:** Institute for Ethics, History and Philosophy of Medicine, Hannover Medical School, Carl- Neuberg-Str.1, 30625 Hannover, Germany

**Keywords:** Intersectionality, Bioethics, Systematic review, Social justice in health care, Feminist bioethics

## Abstract

**Background:**

Intersectionality is a concept that originated in Black feminist movements in the US-American context of the 1970s and 1980s, particularly in the work of feminist scholar and lawyer Kimberlé W. Crenshaw. Intersectional approaches aim to highlight the interconnectedness of gender and sexuality with other social categories, such as race, class, age, and ability to look at how individuals are discriminated against and privileged in institutions and societal power structures. Intersectionality is a “traveling concept”, which also made its way into bioethical research.

**Methods:**

We conducted a systematic review to answer the question of where and how the concept of intersectionality is applied in bioethical research. The PubMed and Web of Science databases were systematically searched and 192 articles addressing bioethical topics and intersectionality were finally included.

**Results:**

The qualitative analysis resulted in a category system with five main categories: (1) application purpose and function, (2) social dimensions, (3) levels, (4) health-care disciplines and academic fields, and (5) challenges, limitations, and critique. The variety of academic fields and health-care disciplines working with the concept ranges from psychology, through gynaecology to palliative care and deaf studies. Important functions that the concept of intersectionality fulfils in bioethical research are making inequities visible, creating better health data collections and embracing self-reflection. Intersectionality is also a critical praxis and fits neatly into the overarching goal of bioethics to work toward social justice in health care. Intersectionality aims at making research results relevant for respective communities and patients, and informs the development of policies.

**Conclusions:**

This systematic review is, to the best of our knowledge, the first one to provide a full overview of the reference to intersectionality in bioethical scholarship. It creates a basis for future research that applies intersectionality as a theoretical and methodical tool for analysing bioethical questions.

**Supplementary Information:**

The online version contains supplementary material available at 10.1186/s12910-024-01057-5.

## Background

Intersectionality is a concept that originated in Black feminist movements in the US-American context of the 1970s and 80s, particularly in the work of feminist scholar and lawyer Kimberlé W. Crenshaw [26]. The historical origin goes back further to Patricia Hill Collins [55] and the Combahee River Collective [25], who criticised White mainstream feminists for making Black women invisible in their struggles. They highlighted the interconnectedness of sexuality and gender with other categories, such as race, class, age, and ability and wanted to be visible in their lived realities as Black queer women with the complex experiences of oppression [25]. Until the late 1990s, the discourse on intersectionality was dominated by the “Big Three” of gender, race, and class, which were analysed as a triple oppression of women. There were already increasing doubts about the additive quality of these categories from the late 1980s onwards, articulated especially by Crenshaw [26, 27]. She highlighted that the reality of discrimination against Black women in the US is much more complex, and that it is defined by “intersecting oppression” [27]. In her works, she harshly criticises the missing and ineligible legal protection for Black women. In terms of the use of dimensions such as gender, race, and class, Crenshaw states that one should not take an “additive account”. This would imply an addition of single axes such as race, gender, and class - e.g. that a Black woman is discriminated against on the basis of her sex and race, and therefore experiences double discrimination compared to a white woman. Rather than that, one should look at complex overlays and interactions of different social dimensions, which vary according to social, historical, and geographical contexts and cannot be fully distinguished from one another. Different axes, such as gender or age, then form a matrix of domination [56] and lead to “specific forms of complex disadvantage” [5], called “complex social locations” [105].

Intersectionality aims to create an awareness regarding how groups of people and individuals are affected by their social position in different systems and structures of power, such as laws, policies, governments, religious institutions, and the media and their maintenance on various levels [114]. These power relations are not mutually exclusive, but instead build on each other and affect all aspects of the social world [57]. As Patricia Hill Collins and Sirma Bilge [57] frame it: “Intersectionality is a way of understanding and explaining complexity in the world, in people and in human experiences”. These experiences are shaped “by the interaction of social locations (e.g. ‘race’/ethnicity, indigeneity, gender, class, sexuality, geography, age, disability/ability, migration status, religion)”, through which “interdependent forms of privilege and oppression shaped by colonialism, imperialism, racism, homophobia, ableism and patriarchy are created” [52]. Hill Collins and Bilge recognise the unlimited ways in which intersectionality is used and develop the following six core ideas to grasp intersectionality: Social inequality, intersecting power relations, social context, relationality, social justice, and complexity [57]. According to Patricia Hill Collins [56] intersectionality is simultaneously (1) a field of study, (2) an analytical strategy, and (3) a critical praxis, which reflects its initial connection to activism and its “transformative potential” [14]. As a field of study (1) we examine the development, topics, boundaries, and debates concerning intersectionality [12]. Intersectionality as an analytical strategy (2) asks “how intersectional frameworks provide new angles of vision” for social inequality [56]. Intersectionality as a critical praxis implies looking at certain social actors and how they use intersectionality to promote social justice. In the context of health care, Bowleg [14] defines intersectionality praxis as “the practical application of intersectionality to facilitate equitable health policy and practice for intersectionally marginalized groups”.

Originating in feminist and antiracist scholarship, intersectionality is often described as a “traveling” concept or theory [22], which has changed over time and is currently applied in different disciplines and manifold ways. Various scholars and disciplines have taken up key ideas of intersectionality, leading to a wide scope of approaches considering various axes of analysis [12, 51]. Walgenbach [107] highlights that the paradigm of intersectionality is work in progress, which aims to open up new research perspectives while always focusing on the analysis of power structures and the aim of making social change. The term “intersectionality” has been widely taken up by scholars, policy advocates, practitioners, activists, and grassroots organisers to inform their research, work, or campaigning [52].

Looking more specifically at health, health care, and medical research, Olena Hankivsky [51] highlights that intersectionality can be used to recognise the specific lived experiences of people, and thereby “aims to improve our understanding of the complexity of social processes and oppressive vectors affecting illness experiences” [23]. The examples above already shed light on how intersectionality is increasingly being applied and used in various fields of health (care) research from health equity research [69] through public health [14] to (counselling) psychology [48] and quantitative psychological research [38]. Dhamoon and Hankivsky [33] argue that the use of intersectionality in health research makes “a concrete difference to the understanding and interrogation of a variety of health issues” such as mental health, violence against women, and HIV/AIDS, as well as the access to and quality of health-care services. It is argued, that intersectionality has the potential to add to biomedical approaches to health, existing tools of analysis, such as gender-based research, and social determinants in health-care research [33]. Furthermore, intersectionality can be used to not only look at the experiences of multiply marginalised groups and identities, but also understand the interlocking social structures and power relations reproducing social inequalities in health [79].

Bioethics constitutes an interdisciplinary research field addressing ethical issues in clinical practice, biomedical research, and public health [30]. Scholars of bioethics address questions of social justice, discrimination, and other ethically debatable practices in manifold ways. Academic contributions to bioethics are published in academic journals and books dedicated to the field, as well as in publications of specific clinical and public health fields, social sciences, or health systems research. Academic debates may be more likely to take place in specific disciplinary proceedings, and ethical contributions in specific areas of application in other journals may increase the visibility of bioethics and support ethical practice. In addition, there are mainstream approaches, such as principlism, and alternative approaches, such as feminist ethics and care ethics, widely used in fields such as nursing – with our open approach we aim to include all of them with respect to their relation to intersectionality.

Various scholars criticize that bioethics do not sufficiently address issues of social justice, including intersectional perspectives [32, 42, 86]. This criticism comes e.g. from feminist perspectives arguing that conceptions of justice need to address “real-life contemporary forms of structural injustice” and look at how forms of oppression and domination are also sometimes exacerbated by health policies and practices in healthcare [42]. De Proost [32] adds to such feminist perspectives with her critique of principlism as focusing on individuals instead of power relations and social justice, arguing that an intersectional approach could inform theorizing. Further criticism comes from Black Bioethics, which could “help reshape how bioethicists apply basic principles like justice” to consider Black peoples lived realities in a complex, intersectional way [86].

The use of intersectionality as a theoretical and methodological background and new research perspective has not yet been considered in a systematic way in bioethical research. However, narrative, scoping, or systematic reviews of intersectionality can be found in other disciplines and might offer inspiration for the use of intersectional perspectives in bioethical research. Siira et al. [96], for example, did a systematic review on intersectionality in nursing research and highlight that there is a need for “robust and clear framing of how the concept of intersectionality is defined” - this might also be true for intersectionality in bioethics. Some other reviews focus on concrete methods and methodology, such as intersectionality in quantitative research [11], or highlight a concrete approach such as community-based participatory research as a well-suited method for intersectional perspectives [58]. Moreover, different reviews highlight that intersectionality should be incorporated in the whole research process, from conceptualization and development of research questions, researched population/sample, to analysis, and to the way results are reported [29, 74, 102].

As one of the first attempts of considering intersectionality for bioethics discourse and practice, Wilson et al. show in their influential article on intersectionality in clinical medicine how an intersectional framework can be applied in the clinical context, looking at patient-physician interactions while considering how power shapes “institutions and clinical priorities” [113]. Barned et al. [9] focus on practical and ethical implications of intersectionality on the institutional level and on structures to see how such institutions might perpetuate social inequities. They argue that it is important to reflect “on the complexity of health care systems and their embeddedness in broader social contexts” [9]. Concerning the ways of applying intersectionality in bioethics, Barned et al. highlight the need to consider “bioethicists’ own positionality and underlying epistemic assumptions about the place of political engagement in ethical reflection and the processes and practices that need to change to support theoretical commitments to overcome bias and promote justice” [9].

Although intersectionality has occasionally been referred to in bioethics e.g. in the context of mental health care or certain social determinants of health such as migration background [72], a systematic overview of its function and use in ethical debates around health and health care is still missing. This systematic review (SR) is, to the best of our knowledge, the first to provide such an overview of the reference to intersectionality in bioethical scholarship. The timeliness and relevance of this overview is further underlined by current (bio)ethical debates e.g. on the treatment of trans people in health care systems [91], on gender and race inequities in the realm of digital health [40], forced sterilizations e.g. of Romani women [3], or questions around surrogacy [43, 63]. Additionally, within the Covid-19 pandemic many health care disparities have been unveiled and inadequacies of current bioethical research have become visible in discussions around social justice [83, 100]. For example, it was clear that sex and gender have an impact on SARS-CoV-2 infections and mortality [15]. However, these factors, as well as race and class, have not been consistently included in research on SARS-CoV-2 [15, 53].

This review aims to serve as an inspiration and provides examples for where and how researchers may use the concept. Moreover, it enables bioethics researchers to develop a reflexive attitude toward their own work. The central objectives of this SR are to provide an overview of where and how the concept of intersectionality is applied in bioethics literature and to identify strands of these debates where intersectionality is mentioned and how it is defined. Accordingly, the SR creates a basis for future research that applies intersectionality as a theoretical and methodical tool for analysing bioethical questions. It may help future researchers to see where and how the concept of intersectionality has already been used, and what they could use it for in their own research in the field of bioethics.

## Methods

Systematic reviews are a well-established method for synthesizing health sciences-related information in a systematic, transparent, and reproducible manner [60]. The aim is to ensure the comprehensiveness of the information given, reduce biases, and inform health-care decisions and policies [21]. The SR methodology was adopted and further developed to account for the peculiarities of bioethical research, which is characterised by a close connection between normative and empirical research questions. Different types of SRs in bioethics can be distinguished, such as those of ethical conclusions, arguments, issues, concepts, values/norms/principles, or recommendations [76]. Even if SRs constitute a rather new methodological trend in bioethics [77], some publications underline the need, for example, for SRs of reasons and their benefits and methodological value for bioethics to inform decision-making [98].

This review aims to serve as an inspiration for where and how bioethicists can use the concept of “intersectionality”, by providing concrete examples from existing publications. This project encompasses empirical, non-empirical (as not essentially data-generating), and normative literature (aiming for moral evaluation or value judgment). The aim is to provide a broad overview of a still rapidly developing and new field of research where intersectionality is used to address health-related (bio)-ethical questions. The PRISMA-Ethics (‘Preferred Reporting Items for Systematic Reviews and Meta-Analyses’) guidelines [60] were followed to ensure the full reporting of all relevant aspects. A study protocol was registered with the Open Science Framework (https://osf.io/uw4xm/) prior to conducting the SR.

### Search strategy and eligibility criteria

We used the PubMed and Web of Science databases to identify relevant publications. We chose PubMed as a widespread database in the health and medical sector and Web of Science to include interdisciplinary research results on intersectionality in health-related research on ethical questions, including the social sciences.

To develop the search terms, two thematic clusters were derived from the research question: (1) *intersectionality* and (2) *bioethics*. We tested different combinations of search terms out of the clusters in the two databases. To exclude non-relevant hits, *health care context* was manually applied as an eligibility criterion. This includes health care research as well as public health practice. Thus, topics such as climate change and gender or feminist literature were excluded.

**Fig. 1:**
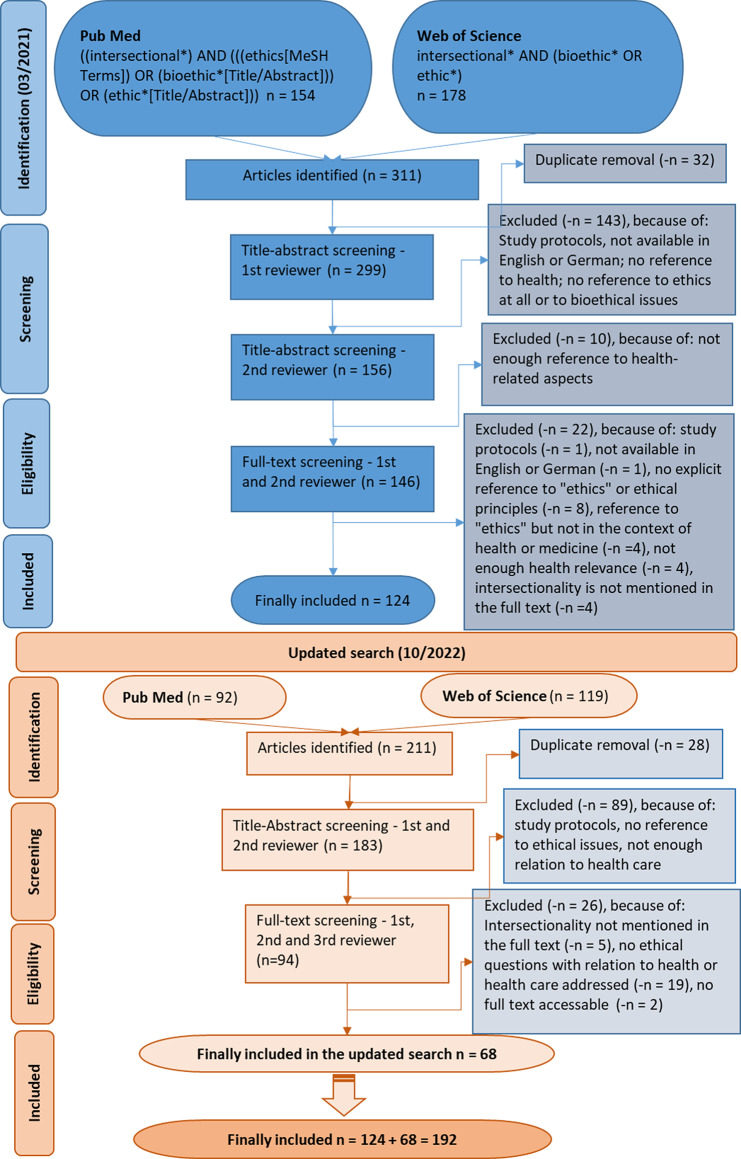
Flow-chart illustrating the in- and exclusion of articles

First, the PubMed search term was developed. Second, the search term for Web of Science was derived from the initial search term, both using the Boolean operators “AND” and “OR” to connect the terms. The final search terms are displayed in Fig. 1. The database search took place in March 2021 and we updated it in October 2022 because we suspected an increase in relevant publications. Therefore, the time period considered in this review was limited from 1989 (when Crenshaw first coined the term intersectionality) until October 2022. The articles underwent an examination according to predefined inclusion and exclusion criteria in a two-stage screening process: title-abstract and full-text screening. To remove duplicates and support the screening process we used the software EndNote.

The following inclusion and exclusion criteria were applied:

Inclusion criteria:

- terms “ethics” or “bioethics” or reference to ethical questions.
- explicit reference to the concept of “intersectionality”.
- health care-related articles.
- language: English or German.
- articles in international, peer-reviewed journals.

Exclusion criterion:

- study protocols.

In this review the term “bioethics” refers to clinical, research, and public health ethics and related fields. Regarding the inclusion of publications in the review, it was decisive whether the authors labelled their research and publication as a contribution to the *ethical* debate. The (methodological) quality of the articles screened did not serve as a criterion because of missing standards for quality appraisal in SRs in bioethics [75]. We did not conduct a reference check due to the manageability of the scope of data.

### Article selection and screening

The first two authors screened all titles and abstracts in the initial databank search independently and applied a staged approach to discuss and consent to every 20–30 articles. The first author (L.B.) conducted the full-text screening and more than 10% (*n* = 15–30) of the articles were randomly cross-checked by the second author (H.K.). Any disagreement about whether a full text should be included in the review was resolved by consensus. Difficult cases were discussed further with the third author (S.S.). A total of 124 articles were included through the initial search in accordance with the eligibility criteria mentioned earlier (see Fig. 1).

We updated the search in October 2022 - also see Fig. 1. The first two authors conducted the title-abstract screening of this update and all the authors screened the full-texts. Finally, another 68 articles were included in the updated search, leading to the final inclusion of a total of 192 articles (see Supplement 2) in this review (see Fig. 1).

All authors are White, cis, and able-bodied, and work in the Institute for Ethics, History and Philosophy of Medicine at Hannover Medical School. Lisa Brünig has an academic background in political sciences and diversity research, Hannes Kahrass is a physiotherapist and has an academic background in Public Health. Sabine Salloch is a full professor of medical ethics with academic backgrounds in medicine and philosophy.

### Qualitative analysis and system of categories

Each article was analysed in full text according to the principles of qualitative content analysis (QCA) in reference to Kuckartz [66]. Passages were coded that included information about how and where intersectionality as a concept was used and/or mentioned. We assigned each quote to one or more codes. We used the software MAXQDA (2020) to support the coding, as it makes the structuring and analysis of textual documents clearer and more transparent, and helps to manage large quantities of qualitative data. The QCA is an interpretative evaluation method for processing qualitative data [67]. An important principle is that the analysis starts from everyday processes of understanding and interpreting linguistic material, and its rules are based on psychological and linguistic theory of everyday text comprehension. What is relevant here in terms of research pragmatics is that this method can be applied to larger amounts of text. Kuckartz [67] highlights that whichever variant of QCA is used, the focus is always on working with categories as tools for analysis and developing a category system (also called: a coding frame). The individual categories represent aspects of analysis that are extracted from the material and arranged non-hierarchically, but instead complement each other and address the key aspects to answer the two-sided research question [66]. Concerning the development of categories, Kuckartz [67] describes three main procedures: (1) concept-driven (deductive), (2) data-driven (inductive), and (3) a mix of deductive and inductive development of codes. We used a mixed deductive/inductive approach in the coding procedure in this review.

The analysis followed the general workflow of a QCA, which operates on a circular basis with categories and subcategories and the coding of data taking place in several cycles [66]. Kuckartz [67] generally describes five phases of QCA: (1) reading the data intensively, (2) building the coding frame, (3) coding the data, (4) analysing the coded data, and (5) presenting the results. In the present review, at first, categories were developed in a deductive/concept-driven way as a coding frame and assigned to individual text passages [66]. Table 1 shows the list of all deductive categories. The “three level” categories are based on Hankivsky’s work [52], as she highlights that intersectionality can be used to understand processes on and between different societal levels, including a micro, meso, and macro level. The category “referencing of the concept” is based on research showing that the concept of intersectionality has been increasingly picked up by more and more disciplines with the risk of obscuring the origin of the term in Crenshaw’s work [22]. The category “mention or definition” is based on the criticism that intersectionality is often only used as a “buzzword” [31]. The categories patient-physician interaction, access to health care, research, and education are inspired by the work of Wilson et al. [113], who discuss in which ways intersectionality can be valuable in the clinical context, focusing on patient-physician relations. Some of the deductive categories such as the application purposes as well as the medical disciplines and the different actors it could be applied to are related to the main research questions of this systematic review.

**Table 1:** List of initial deductive categories

WHERE?	
	- Patient-physician interaction* - Access to health care* - Research* - Education*
	- Medical discipline (including public health, nursing, psychology)
	- Applied to the patient* - Applied to the medical professional/personnel* - Applied to the researcher*
HOW?	
	- Referencing of the concept* • To Crenshaw • To others? Whom?
	- Mention or definition • Defined HOW?
	- Application purpose/Function • To determine/describe a phenomenon • To explain a phenomenon • To resolve a problem • To develop something further

* These categories were not included in the list of the final main categories for analysis (see Supplement 1)

During the coding process, these deductive (concept-driven) categories were inductively (data-driven) supplemented by further subcategories (see Supplement 1) while some of the deductive categories in Table 1 were deleted because they seemed less relevant during the coding process. The category building was performed concurrently with coding and structured along the two central aspects within the research question, namely the questions of “where” and “how” intersectionality is applied within bioethical research. Regular team discussions took place on the assignment of the codes, the further development of the code list, and the system of categories derived. All three authors discussed and agreed on the assignment of codes to certain unclear text passages in regular meetings. Therefore, we developed the whole system of categories cooperatively.

The data analysis ultimately resulted in a system of categories, which represents the central instrument of the analysis and serves to systematize the content relevant to the research question. We completed the deductively created categories inductively in the coding process and then compressed, rearranged, and finalized the category system. In the following step, we selected five main categories for further analysis (see Supplement 1): (1) application purpose and function; (2) social dimensions; (3) levels; (4) health-care disciplines and academic fields; (5) challenges, limitations and critique. Only these five main categories were coded in the articles identified through the updated search. Due to the richness of the qualitative data, only the results derived from these five categories are reported in the following.

## Results

**Fig. 2:**
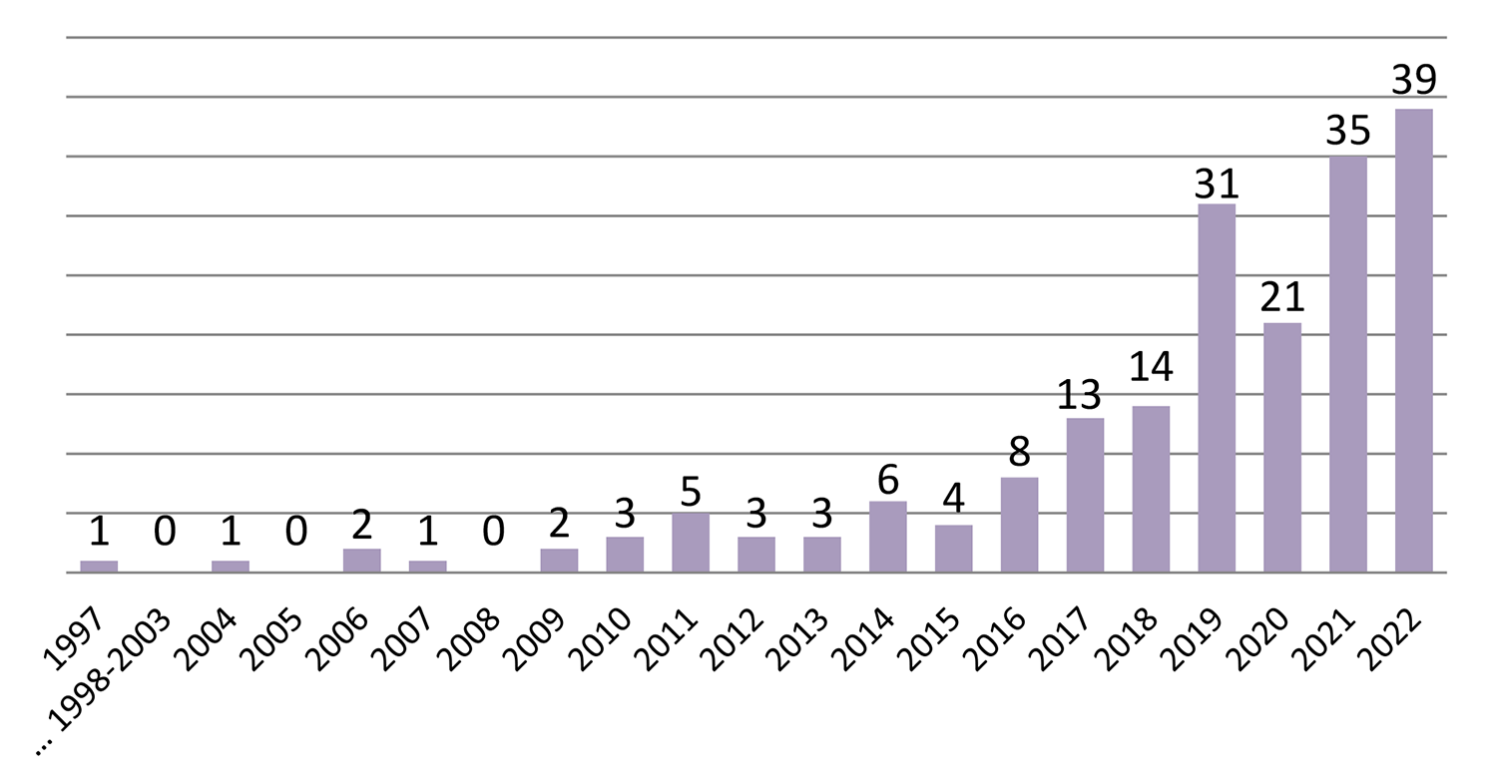
Number of included articles per year

**Fig. 3:**
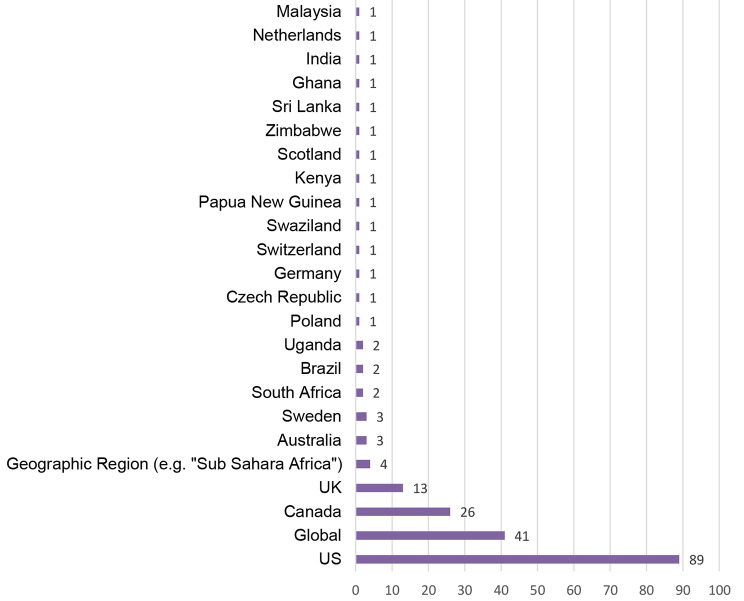
Geographical scope of articles (context of the analyses)

More than half (52%; *n* = 100) of the 192 articles included are theoretical and 48% (*n* = 92) are empirical papers. Of the empirical studies, 95% (*n* = 87) followed qualitative approaches, 3% (*n* = 3) followed quantitative approaches, and 2% (*n* = 2) followed mixed-methods. When looking at the number of articles per year included, there has been a slow increase in publications over the years from one in 1997, to eight in 2016 and to 14 in 2018 (see Fig. 2). What follows is a greater increase with approximately 80% (*n* = 154) of the articles in this review published in the four years from 2019 to 2022. This mirrors the expectation that the term and concept of intersectionality has been increasingly taken up in bioethical research since 2019 – with the trend rising. Additionally, the articles included cover a broad geographical scope. While most of the research is based in a US-American and Canadian context, some of the studies are from African countries, such as South Africa, and from European countries, such as Poland. Figure 3 illustrates all countries covered in this review based on the contexts the respective authors mention in their articles. These contexts do not always match the affiliation of the lead authors but we found it important to show the contexts the results refer to. In 41 articles, the results broadly refer to a global context.

### “Where” the concept of intersectionality is used

The first part of the research question asks *where* the concept of intersectionality is applied in research on bioethical questions. Two main categories were built to analyse this distribution: the various health-care disciplines and academic fields in which the authors of the selected articles locate their research were quantified, and the three levels (micro, meso, and macro) and their relevance for an intersectional perspective were distinguished.

#### Health-care disciplines and academic fields

As a result, intersectionality is used for ethical issues occurring in a broad variety of health-care disciplines and academic fields throughout the articles included in this review (see Table 2).

**Table 2:** Health-care disciplines and academic fields

Health-care disciplines			Academic fields		
Discipline	Number of codings/number of articles	Percent of all articles included	Field	Number of codings/number of articles	Percent of all articles included
Mental Health/Psychology	943/74	38,5%	Medical/Bioethics	588/62	32,3%
Nursing and Nursing Research	326/21	10,9%	HIV/AIDS	217/17	8,9%
Gynaecology/Reproductive & Sexual Health	307/27	14,1%	Feminist Perspectives	124/52	2,7%
(Social) Gerontology	23/6	3,1%	Public Health	104/31	16,1%
Palliative Care	11/2	1%	Global/International Health	77/9	4,7%
Genomic Medicine	8/2	1%	Trans-focused research	50/3	1,6%
Pediatrics	6/3	1,6%	Disability Studies	45/15	7,8%
Cardiovascular Medicine	2/1	0,5%	De/postcolonial perspectives	36/8	4,2%
			Social Work	32/5	2,6%
			Health Services Research	31/7	3,7%
			COVID-related	29/14	7,3%
			Gendered Violence (prevention)	16/5	2,6%
			Medical Humanities	12/1	0,5%
			Philosophy	12/3	1,6%
			Health Policy Research	11/2	1%
			Gender Studies	5/5	2,6%
			Deaf Studies	2/1	0,5%
			Medical Sociology	6/4	2,1%

The disciplines and fields captured in the system of categories are material-based and refer to the terms and descriptions that authors use themselves to classify their research. Therefore, the disciplines and fields are based on categorisations in the abstracts and full texts. A total of 26 categories arose: 8 health-care disciplines and 18 academic fields. The health-care disciplines include highly institutionalized health-care disciplines, such as psychology (943 in 38,5% of the articles), gynaecology (307 in 14,1% of the articles), gerontology (23 in 3,1% of the articles), and paediatrics (6 in 1,6% of the articles) (see Table 2). On the other hand, other academic fields not directly linked to health care are included, such as disability studies (45 in 7,8% of the articles), philosophy (12 in 1,6% of the articles), gender studies (5 in 2,6% of the articles), and deaf studies (2 in 0,5% of the articles). These fields appear because researchers locate their own research within these fields or reference to gender studies and gender theories as beneficial, for example, for public health [84] and for answering bioethical questions. In addition, the system of categories includes the identification of articles focused on a specific research field, such as HIV/AIDS, COVID-related studies, or research on trans people and their health (care). The articles included also explicitly located themselves in the context of de- or postcolonial studies (36 in 4,2% of the articles). As visualised in Table 2, mental health/psychology, medical/bioethics, and nursing and nursing research are the fields that were coded the most within this review. Therefore, concerning bioethical questions, these seem to be the (research) areas where the term “intersectionality” has been most frequently used until now. This scope of fields and disciplines is also mirrored in the top 10 journals, included in this systematic review. Table 3 illustrates the journals most articles were published in as well as the number of articles from these journals that have been included in this review.

**Table 3:** Top 10 journals

	Journal	Number of articles included from this journal
1	American Journal of Bioethics	13
2	American Journal of Psychology	7
3	ANS Advanced Nursing Science	9
4	The Arts in Psychotherapy	6
5	Gender Place Culture	5
6	Journal of Counselling Psychology	5
7	International Journal for Equity in Health	4
8	Social Science and Medicine	3
9	Journal of Bioethical Inquiry	3
10	The Canadian Journal of Nursing Research	3

The two following examples will further clarify the use of intersectionality in different disciplines and fields: In regard to intersectional perspectives concerning ethical questions in gynaecology and reproductive/sexual health, certain topics occur as those most frequently addressed, such as assisted reproduction and surrogacy, lactation care, obstetric care and violence, birthing, abortion, contraception, and sterilization (see Supplement 1). Most of the studies included from this field are qualitative studies (63 %; *n* = 33); the rest are theoretical papers. Interestingly, many of these articles work with the concept of reproductive justice, which is an “increasingly popular framework for understanding broad-ranging inequities relating to reproduction” originally coined by the SisterSong Women of Color Reproductive Justice Collective in the USA [74]. Therefore, the concept is based on an activist movement that follows an intersectional perspective focusing on questions such as disparities in abortion rates and the influence of “interactive effects of race and class” [85]. The concept of reproductive justice thus exhibits a kind of “intersectional extension” when incorporating reproductive rights and health, including aspects of (unintended) pregnancy, contraception, abortion, birth, family formation, and parenting in safe and healthy environments. Price argues that “a singular focus on abortion rights neglects how race, ethnicity, class, sexuality and other markers of difference are implicated in reproductive rights for many women” [85].

As a second example, it is interesting that mental health and psychology play a dominant role in the sample of publications analysed. The subcategories demonstrate (see Supplement 1) that the focus of the articles using intersectionality included is on certain health conditions, such as trauma and post-traumatic stress disorder, autism, schizophrenia, and addiction. On the other hand, the system of categories displays certain areas, such as mental health nursing, education and training in psychology, as well as the often-coded area of psychotherapy and counselling psychology. An elaborated argument for incorporating intersectionality into mental health issues can be found in the work of Rosenthal [90]. She argues that

there are large bodies of existing evidence about the adverse effects of both interpersonal and structural oppression, inequality, and stigma across the spectrum of human experience and behavior […], suggesting these issues fall squarely within the realm of psychology across subfields, populations, and specific phenomena of interest for psychologists [90].

A closer look at the subcategory of psychotherapy and counselling psychology shows that marriage and family therapy and creative arts therapies are especially dominant topics in research from an intersectional viewpoint (see Supplement 1). Some authors, for example, focus on the benefit of intersectional perspectives in creative arts therapy to reconsider its foundation “to open a conversation about intersectionality and the ethics of care” [101]. Van den Berg and Allen voice concern about the “overwhelming Whiteness of art therapy” [103] while Wright and Wright [117] qualitatively analyse the experiences girls and young women living with violence-based trauma have in art therapy in the United Kingdom.

Insight into the discipline of paediatrics shows that different topics are discussed here in relation to intersectionality. Bannink Mbazzi and Kawesa [8], for example, underline the importance of the intersectionality of family-centred care, poverty, and neo-colonialism when working with children with neurodevelopmental disabilities in the Global South and aiming for improvements in the provision of health care. Another topic occurring in this field is the consideration of family immigration status as an intersectional social determinant of health interacting with other determinants such as race, religion and language [72].

To give one last example for this category, the article by Sikka [97] in research concerning palliative care casts a feminist perspective on the topic of medically assisted dying and the ways in which (structural) barriers influence access to it for marginalised groups. Another article from the German context [79] shows how an intersectional perspective on end-of-life care policy discourses reveals that a critical theoretically grounded notion of difference needs to be incorporated into relevant ethical principles of end-of-life care.

#### Levels

As visualised in Fig. 2, this category is concept-driven and based on the definition of the concept of intersectionality, which includes the tenet of multilevel analysis across questions of identity, representation, and structure [33, 115]. As Hankivsky points out:

Intersectionality is concerned with understanding the effects between and across various level in society, including macro (global and national-level institutions and policies), meso or intermediate (provincial and regional-level institutions and policies), and micro levels (community-level, grassroots institutions and policies as well as the individual or ‘self’) [52].

The most prevalent level in the research included is the individual level, followed by the structural level, as reported in Supplement 1. However, the institutional level is mentioned in fewer articles and therefore, garners less attention in bioethical research, as shown in this review.

The micro level focuses mostly on individual experiences. Many arguments on this level include aspects related to “identity”, meaning the intersectionality of multiple identities or identity categories such as gender and race, which shape individual lived experiences and create unique forms of oppression [110]. These categories themselves are defined as “social constructions”, often binary, while intersectionality “allows for the view of each identity as being on a continuum that is fluid, constantly shifting and adapting to social contexts” [80]. On the micro level, a focus is on the needs and barriers that individual people or communities face in regard to access to health care, for example, related to “multiple axes of identity that influence personal HIV risk” [78]. Other examples are analyses of the role and responsibility of the individual physician to improve, for example, the health of trans people [114]. Questions are addressed such as how an individual is socially located within society or within the hospital itself. This “location of self” is also referred to in relation to researchers and therapists and their need to self-reflect to follow an intersectional practice. Watts-Jones, for example, analyses how the transparent reflection of her own privileges and discriminations in society compared to those of her patients in therapy influence the therapeutic relationship – she calls this “opening the door to dialogue on intersectionality in the therapy process” [108]. Rogers and Kelly [88] criticise how the intersection of different social groupings at the level of the individual are rarely considered or addressed in health research. They argue that people instead are being ranked according to only one aspect, for example disabled people are considered more vulnerable than people from ethnic minorities.

Individuals on the micro level can be seen as a starting point of looking at their positioning in institutions and power structures. Therefore, the meso level in this review focuses on power structures in health-care institutions, such as hospitals and care facilities, as well as within academia and education. One example is the profession of nursing, where van Herk et al. [104] also apply an intersectional lens to nursing education and research. The authors argue that an intersectional paradigm paying attention to unequal power relations would be helpful in “nursing organizations and the institutional settings in which nurses work” to act out “our ideals of social justice in a practical and meaningful way” [103]. Weitzel et al. [109] also argue that nurses could act as allies against racism in the respective institutions. Another example on the meso level is the focus on certain health-care sectors, such as the Swiss health-care sector with its increasing specialisation of physicians and interprofessional collaboration [4]. Further main topics are the economisation within the health-care sector and neoliberal market logics [10], the depersonalization of health care [44], and feminisation of the medical profession [4]. Authors such as Barned et al. [9] highlight the role of institutions in perpetuating social inequities. Asakura and Maurer [6] call for challenging institutional practices that perpetuate the marginalisation of certain clients or patients. To give a more concrete example, Muntaner and Augustinavicius [82] discuss hiring practices in health-care institutions and argue that, first, doctors from the class, gender, and race of the communities they serve need to be recruited before good clinical practice can be implemented.

On the macro level, we find the evaluation of certain health-care policies, laws, legislation, and the resulting inequities. Examples for these are anti-ableist policy recommendations by Fine [41] or analyses of forced sterilization policies by Albert and Szilvasi [3]. Furthermore, the role of health authorities, such as the American Psychological Association, and international health organisations, such as the World Health Organization, and their actions in the context of discrimination through policies are analysed [16]. Overall, the focus of the macro level is on power structures as an essential characteristic of intersectional approaches. Williams [112], for example, looks at how intersecting systems of privilege and oppression, such as global capitalism, neoliberalism, patriarchy, imperialism, and postcolonialism shape the transnational political economy of care with certain practices of care labour. When using an intersectional framework, researchers must consider structural discrimination and its effects, for example, on the health of lesbian, gay, bisexual, trans, queer, inter-, and asexual (LGBTQIA+) people [49]. Therefore, the “fundamental causes” of social inequalities in health are taken into consideration, such as poverty and social discrimination [13, 71]. The last aspect mentioned on the macro level concerns historical forms of discrimination, such as White supremacy [46]. It is deemed relevant to consider the effects of being a member of a marginalised community, which has experienced enslavement, attempted genocide, mass incarcerations, and neocolonialism in the sense of racial trauma in regard to trauma recovery [17].

Many authors highlight that these three different levels cannot be separated when using an intersectional perspective. One example is the article by Macleod on the concept of reproductive justice, where the three levels are described as inseparable and “as the intertwining of individual and social processes” [17]. Returning to the study by van Herk et al. on nursing, the following quote exemplifies how intersectionality as interlocking systems of oppression highlights the linkage of individual, institutional, and structural levels:

Often in the current healthcare environment of efficiency, with the limited time and resources within which many nurses work, it feels like there is little time to really understand our patients. However, we need to question a system that puts more emphasis on money and efficiency than giving us the time, education, and resources we need as nurses to be able to provide the type of care that not only have we been trained to do and which our profession requires of us, but which as dedicated and compassionate human beings we want to be able to provide [104].

### “How” the concept of intersectionality is used

The second part of the main research question asks *how* the concept of intersectionality is used in bioethical debates. On this second axis, three main categories were developed to answer this question, including (1) the different social categories or dimensions, (2) the functions and application purposes of the concept of intersectionality, and (3) the challenges, such as implementing the concept in clinical settings, limitations, and critique of the concept.

#### Social categories/dimensions

**Fig. 4:**
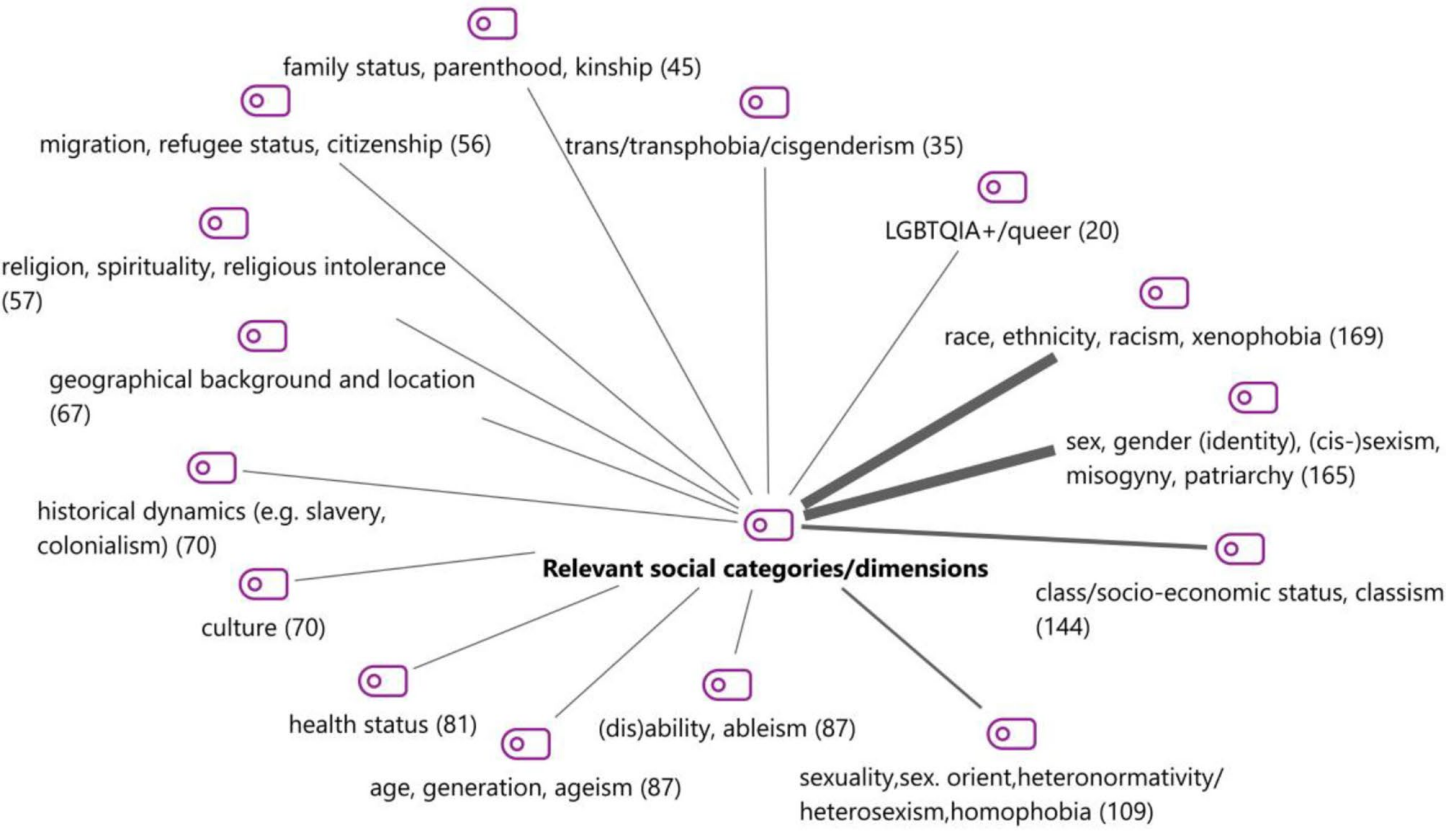
Social categories/dimensions of intersectionality (number of articles)

A total of 22 different social categories emerged during category development (see Supplement 1), including age, body, language, migration status, family status, education, professional status, and caste. Different terms are being used for the issues considered in intersectionality, such as “categories”, “dimensions”, or “aspects of identity” [36], while others talk about the “intersection of social stratifiers” [7]. Figure 4 visualises the 15 most frequently coded social categories, including subcategories. Overall, the category system contains 48 categories, including 22 main and 26 subcategories.

As expected, the established triad of race, class, and sex/gender [64] was most frequently used in the articles included, and can be further stratified into subcategories. The category sex and gender (identity) includes terms for systemic forms of oppression, i.e., (cis-)sexism, misogyny, and patriarchy. The most frequently quoted subcategory is trans/transphobia/cissexism, analysed e.g. in the context of health literacy for LGBT migrants [106] or when it comes to healthcare mistreatment of transgender and gender diverse individuals of colour in the United States [91]. However, aspects concerning intersex people have only been mentioned in two articles (see Supplement 1) in St. John’s article on infant mental health, which shows that intersex infants as well as “gender fluid and nonconforming toddlers are almost entirely repressed in infant mental health discourse” [99].

**Fig. 5:**
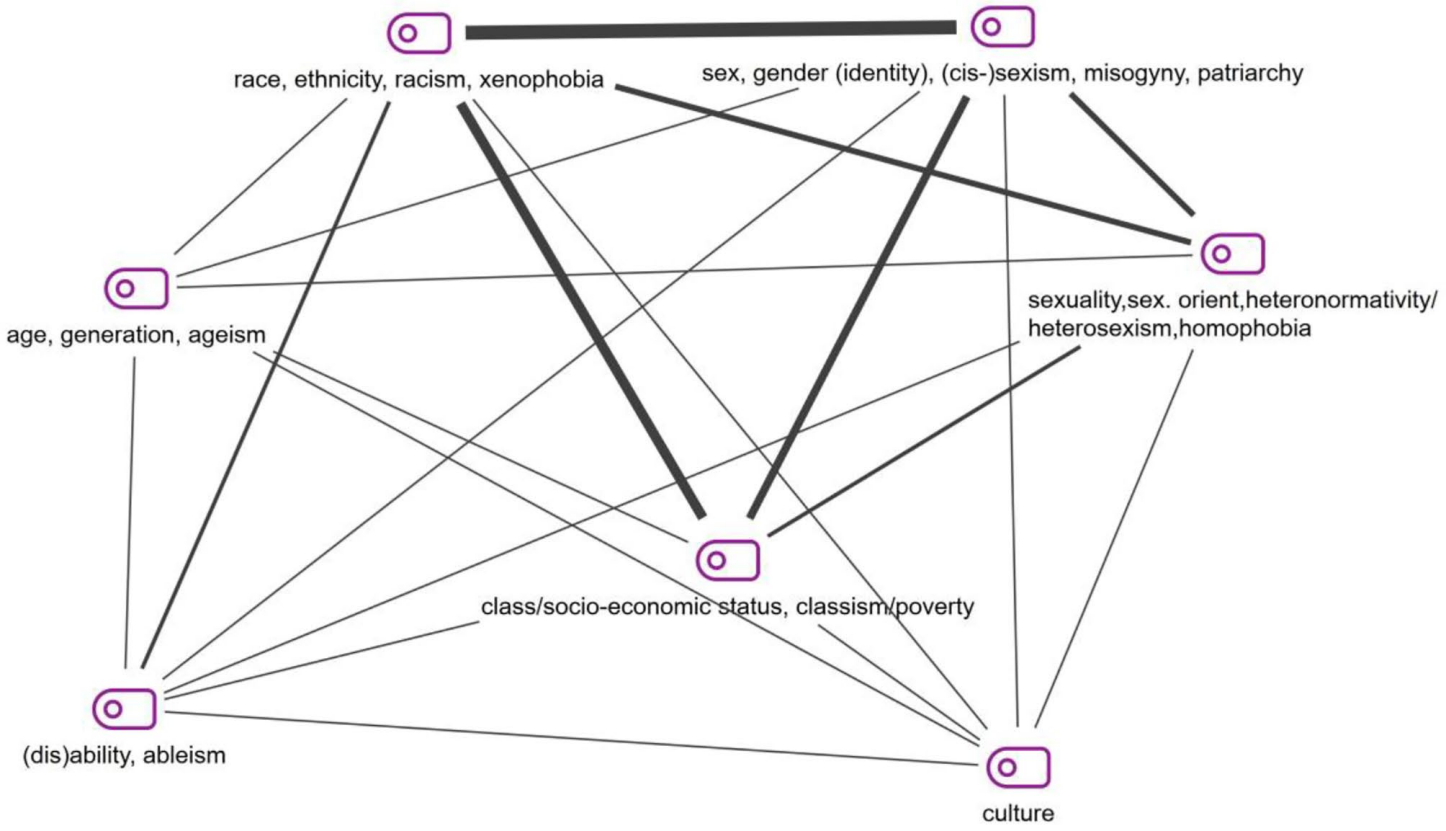
Code relations model

Sexuality/sexual orientation, disability, culture, and age are the most frequently used categories after race, class, and gender (see Supplement 1). In addition, Fig. 5 shows the ways in which the different social categories/dimensions overlap in the analysed articles. This is the so-called “Code Relations Model”. It is created by the software MAXQDA and visualises the simultaneous occurrence of codes in a text segment. The thick lines show the strongest overlap of codes; in this case, it visualises the triad of race, class, and gender. Burger et al. [18] address this triad when analysing public health nursing actions for Black women in the United States, forms of structural violence and ways in which reproductive justice as an intersectional concept can advance health promotion for Black families.

Figure 5 further shows that the dimension of sexuality/sexual orientation often occurs together with class, gender, and race. An interesting example is the study by Burrow et al. [19] on the experiences of queer birthing women in rural Nova Scotia and the influence of heteronormative and homophobic healthcare practices and policies. The analysis focuses on the intersection of queerness and rurality, however more intersections are mentioned, and the sample includes a wide age span, people from working class to upper class as well as White, First Nations, and African-Nova Scotian persons. Another encompassing example is the study by Dubé et al. [35] on trans people, cis women, as well as racial and ethnic minority groups, who are all underrepresented in HIV cure-related research and should be considered.

Issues related to ableism are addressed in articles intersecting with gender, race, and class, but also age and culture. A good example is a review of research ethics literature by Cascio et al. [20], considering how in the case of autism research ethics, sex and gender, race, geography, language, socioeconomic status, age, and level of support needs are considered. The authors highlight that the exclusion of marginalized subgroups such as people with autism “is a major ethical concern” [20]. Another important dimension visualised in Fig. 5 is culture. One example for looking at the intersections of culture, race, and gender is the article by Eagle and Long [36] on psychotherapy in contemporary South Africa and the role, these dimensions play in the relationship between therapist and client in a “post-Apartheid society” [36].

The articles on age in this review mostly discuss ageism together with heteronormativity and address health care needs of older LGBTQIA people [86, 111]. The qualitative study by Cuesta and Rämgård [28] focuses on the intersection of gender and race in the context of power relations in elderly care homes.

On the contrary, professional status, social capital, and caste are the least mentioned categories in this review (see Supplement 1). Professional status mostly occurred in relation to immigration, education, and language, referring to e.g. rights of immigrants in the United States and the ways in which such discrimination increases the risk of illness and lack of access to health care due to financially precarious situations [61], or the situation of Cuban-educated doctors migrating to Florida [50]. The category “social capital” is mostly used in listings of social determinants of health e.g. in the context of Global Health epidemiology [2]. Sometimes it is described as social networks or safety nets which can contribute to health and well-being and are often missing for example for immigrant Latina women as described in the article by Kelly [61]. Caste is only mentioned in one article and analysed in relation to gender. Islam et al. [59] elaborate on the intersection between gender and caste when it comes to increased discrimination of female doctors in India.

#### Functions and application purposes

The main category “functions/application purposes” was created to grasp the core ideas of what intersectionality is used for in the respective research approaches. As visualised in Supplement 1, intersectionality is used in the articles in order to understand and explain a phenomenon, make certain aspects visible, resolve a certain problem, foster interdisciplinary dialogue, and for better health data collection. An aspect that also strongly formed this category is the description of intersectionality with reference to Patricia Hill Collins [56] and Cho and colleagues [24] as a field of study, an analytic strategy, and a critical praxis.

In the articles included, intersectionality is most frequently described as a critical praxis. This incorporates the aspect of working towards justice, solidarity, and representation. Some researchers argue that the concept of intersectionality can be explicitly used to ensure that research results are relevant for respective communities, and thereby foster “a politically engaged bioethics” [47]. An example can be found in Henrickson’s [54] focus on research ethics with gender and sexually diverse persons. The article encourages researchers to use an intersectional perspective in the design and analysis of their data to be able to capture the complexity of participants’ experiences. This is exemplified by a study on the use of PrEP (pre-exposure prophylaxis) among Black men who have sex with men. An intersectional perspective means to “move beyond a biomedical approach to HIV prevention”, consider structural barriers to PrEP uptake such as finances, stigma, or experiences of racism with health care providers and thereby improve respective policies [54].

An intersectional perspective in research is suitable for making analyses deeper and more complex and considering historical contexts, for example, concerning therapy for people from indigenous families who suffer from intergenerational trauma [45]. Gerlach’s [45] analysis in the Canadian context entails a critical reflection of the relevance of neo-colonial practices in therapy with indigenous people to improve their health care experiences, as well as a reflection of own privileges as a white, educated, middle-class therapist. Another aspect of intersectionality as a critical praxis is its potential to inform the development of health-care policies and interventions, such as for meeting mental health needs and the care of trans people [92]. Shelton and Lester [92] specifically analyse the ways in which Black trans women are affected by transphobia, classism, and racism in different regions of the US when it comes to mental health care. Writing from the angle of critical disability studies, Fine [41] demands that when psychologists translate research into policy “we need to center analyses in the lives and experiences of people with disabilities” and consider racism, sexism, and homophobia in the process.

In addition, intersectionality is often described as a tool for critique and researchers’ (self-)reflection. Shimmin et al. [94] develop suggestions and questions for discursive reflection practice for research teams when public partners are involved in their research, using trauma-informed intersectional analysis. They encourage researchers to reflect on their own values, experiences, beliefs, and political commitments in the areas of health that they will be researching.

Another function of the concept that came up in the coding process is the use of intersectionality for theorising the relationship between research and activism. In the context of sexual and reproductive health, Price argues, “as researchers, we can learn from reproductive justice activists” [85]. Regarding the field of psychology, Moradi and Grzanka point out that “counseling psychologists can contribute to translating insights of academic scholarship into activism and social transformation with and on behalf of participants, clients and students” [81]. In their work, these two authors also follow the mentioned definition of intersectionality as a field of study, an analytical strategy, and a critical praxis. Alongside these three aspects, they develop guidelines on “how to use intersectionality responsibly” [81]. They call these guidelines a “call to action” [81] not only for researchers, clinicians, and activists but also for reviewers, editors, and supervisors. Similarly, Rosenthal [90] makes concrete suggestions for individual psychologists on how to apply intersectionality in their research, teaching, and practice, as well as for psychology as a field. She suggests (1) engaging and collaborating with communities in the research process, (2) addressing and criticising societal structures, (3) building coalitions with communities in research, teaching, and clinical work, (4) attending to resistance in addition to resilience, and (5) teaching social justice curricula.

One last exemplary function of intersectionality that is potentially important for future debates in bioethics is the understanding of intersectionality as a form of ethics itself. Grzanka and colleagues suggest that “because of its focus on social action and social justice intersectionality can be thought of as a form of ethics itself and ought to be taught alongside key bioethical principles such as principalism [sic], utilitarianism, and virtue ethics” [47]. This suggestion opens up new debates about the standing and importance of intersectionality in bioethics. Critical voices, however, argue that “the common neutralization of political and epistemic commitments in bioethics […] complicates the integration of intersectionality in traditional bioethics” [9].

#### Challenges, limitations and critique

The last main category answering the question of *how* intersectionality is used in bioethical research concerns challenges and limitations, for example in the implementation and critique of the concept of intersectionality.

One of the most frequently voiced challenges concerns the practical implementation of intersectionality in clinical settings and the everyday work of health-care providers. On the one hand, it is explicitly acknowledged that intersectionality does not offer a “how-to” manual for clinical medicine or a blueprint for ethical action [9]. On the other hand, there is a demand for further discussion on the conditions of its application. Grzanka and Brian highlight that “clinical encounters do not occur in a vacuum” [46], and institutional aspects need to be considered [112]. Lanphier and Anani [68] additionally argue that intersectionality might create the risk within the clinical encounter for clinicians to impose and reproduce biases towards patients. The danger of labelling and putting people into particular groups remains and makes patient narratives necessary [73]. At this point, it is important to view the concept of “bias” itself critically from an intersectional perspective. It can entail the risk of individualization of structural inequalities and of reduction to the personal level of individual attitudes [65]. With reference to Cho, Crenshaw, and McCall [24] structural intersectionality however highlights interlocking oppressions to claim social justice.

Another challenge concerning analysis is the often-posed focus on one main axis, such as racism or sexism, or on one homogenized group identity, which creates gaps in bioethics research [23]. Authors such as Aguayo-Romero [1] and Eilenberger et al. [37] demand that the complexity of analysis should be expanded to also include classism, heterosexism, and other systems of oppression, such as ableism, ageism, cissexism, colonialism, ethnocentrism, nationalism, and colourism. All in all, this broad challenge appears to be about finding a balance between the aim of intersectional analysis to put an emphasis on making power structures and institutionalized forms of oppression visible for more complexity, on the one hand, and the practicability of (especially quantitative) research, on the other hand.

Another fear in relation to the implementation of the concept is an overuse and mainstreaming of intersectionality [4] together with the danger of intersectionality being “coopted, depoliticized and diluted” [81] by focusing only on multiple identities and not addressing structural inequities [90]. Shin et al. [95] argue that the travelling of the concept causes a loss of its transformative potential. The fear is that its origin in an activist, anti-racist, and feminist setting, its contemporary advancements in feminist, gender, and women’s studies, and its focus on power structures could be forgotten.

A common critique is that intersectionality is a vague concept [13] with a lack of clear methodology [112] and is often used as a “buzzword” [31]. We also observed this phenomenon in our data, when certain studies e.g. used the term intersectionality in the title, but never defined or actually adapted it in the full text [3]. However, this ambiguity is also seen as being inherent in the definition of intersectionality and sometimes negotiated as both a weakness and a strength of the concept with opportunities for debate, theorising and research at the same time [13, 31]. Authors such as Muntaner and Augustinavicius [82] call for a clarification of the framework terminology of intersectionality, for example by using more concrete terms to describe social systems. This critique of methodological “murkiness” [13] can be especially problematic in cases of quantitative research because many statistical methods often rely on assumptions of unidimensionality. However, there are some best practices for quantitative projects for example in Price [85], including disaggregating samples by race and gender. Moradi and Grzanka [81] argue that intersectionality does not mandate qualitative methods, but it does mandate critical self-reflexivity also on methods and results, which is common in qualitative research. Researchers need to be aware of their own decision-making process and be able to state why they choose certain intersections, which categories they study, and how these categories align with individuals’ self-perception [45, 61].

Critique concerning the implementation of intersectionality entails that the focus should be not only on marginalisation but also on privileges. Employing the framework just to study marginalised groups is argued to be problematic because privileged groups, such as White, male, able-bodied people remain uninterrogated, which again reinforces their normativity [95].

## Discussion

In the following, we will discuss three aspects emerging from the review findings concerning future research in the field of bioethics and highlight some limitations of this review. When looking at the different academic fields and disciplines identified in the analysis, we notice that intersectionality is not used in all medical and health-care disciplines. Large clinical fields, such as trauma surgery or otolaryngology are completely missing in the review. To better understand this finding, a comparative look at the introduction of intersectionality as a research concept into the fields of gender studies versus bioethics could help. Intersectionality has played a central role in the emergence and development of the field of gender studies. This process also included a politicisation of research and an overlap between scholarship and social activism. Intersectionality significantly transformed the research branch of women’s studies, which, in its beginnings, focused mainly on sex and gender. The introduction of intersectional approaches merged insights from different fields, such as postcolonial, disability, and gender studies. The prominent role of intersectionality in gender studies has interesting implications for its introduction into bioethics, such as the need for self-reflexivity, interdisciplinary cooperation, and the relevance of the interconnectedness between individuals, institutions, and societal power structures. However, there are “disciplinary divides” [62] between academic branches from which intersectionality originates, such as gender studies and bioethical and health research. With reference to Kelly et al. it could be argued that bioethics and health research in general “privileges certain types of knowledge” [62]: “Classifying lived experience and qualitative scholarship as lesser form of knowledge, or excluding them from the diagrams of what ‘counts’ as knowledge contradicts intersectionality” [62]. The origin in research from an intersectional perspective lies in lived experiences and includes an agenda for positive (social) change.

An interesting focus of discussion is the number, combination, and constellation of social categories used in the articles included. As we can see in Figs. 4 and 5, the established triad of race, class, and gender is the focus of research concerning bioethical questions included in this review. However, in the social sciences and gender studies, especially in social inequality research, this triad is argued to be relevant for all modern, capitalist societies in differing specific ways according to the local context. When looking at the way intersectionality developed in social sciences and especially gender studies, we can see that an increasing number of social categories, such as ability, age, sexuality, and religion, have been included in intersectionality research over time. Crenshaw highlighted that: “While the primary intersections that I explore here are between race and gender, the concept can and should be expanded by factoring in issues such as class, sexual orientation and color” [27]. Richter and Kricheldorff [87] e.g. focus on the intersection of sexuality and age and argue that an intersectional perspective can contribute to needs-based care for LGBTQIA senior citizens. Engelman et al. [39] analyse the intersection of disability with gender, age, class (specifically homelessness), professional status, family, education, and race - demanding a disability justice framework for nursing education to improve the quality of care for persons with disabilities.

Moreover, while intersectionality “travels” over time and into different academic disciplines, the meaning of single categories themselves is often debated. The definition and understanding of the category of “race”, for example, differs according to geographical context and historical backgrounds, such as slavery and (neo-)colonial continuities, and is for example related to ethnic, cultural, and religious differences. In our review sexuality, (dis)ability, and age are the categories that are mentioned the most, after gender, race, and class. We therefore argue that these seem to be relevant dimensions in relation to bioethical questions. Examples are questions of autonomy in regard to people with disabilities and their access to certain kinds of health care, such as gynaecological services [116]. Another exemplary topic with a focus on age and sexuality is the question of what assumed heterosexual identity means for patients in geriatric care [110]. The unexpectedly high number of different social categories in this review, such as body and caste, illustrates the openness of the intersectional approach and the possibility of using it flexibly depending on the research interest and historical and geographical context. Some studies e.g. show the intersection of one’s body, with disability, gender, age, and migration [8, 39, 41].

The use of activist knowledge and insight as one of the functions of intersectionality is another important point for discussion. As exhibited in the analysis, the concept of intersectionality can foster politically engaged bioethics and is suited to theorise the relationship between academia and activism. This leads back to the aspect of different understandings of scientific knowledge production itself. An intersectional approach for bioethics researchers necessitates reflection on their own privileges and discriminations within academia and society as a whole. Grzanka and colleagues argue that

intersectionality reminds us that many of the answers to the question of how to do bioethical antiracism will come not from privileged academics, but from the actual individuals, organizations, and advocacy groups already fighting against racism, sexism, classism and other intersecting forms of health inequity in the United States and worldwide [47].

In this context, many authors highlight that intersectionality originates from Black, anti-racist movements and activism, and is, in itself, a “social justice movement” [81]. Therefore, with reference to Crenshaw, researchers should “work to enact resistance and activism as a central rather than ancillary part of research” [81]. To add to this, there is more recent research on the relationship between activist engagement, bioethics, and academia. While Rogers and Scully argue that “bioethics has an inescapable activist element because ethics itself is partisan, in the sense that is always entails a move toward the normative” [89], Draper voices scepticism and argues about “personal and professional risks, as well as rewards, to activism” [34]. Draper designs a taxonomy of impact or engagement activities for academic bioethics as a scale that ranges from little engagement beyond academia to activities defined as activism. An important remark is that it “is not enough to claim that one’s activism is ethically motivated without being explicit about the foundations of one’s motives”, might they be, for example, Catholic, libertarian, or feminist. Moreover, we need to consider the national and institutional contexts when estimating the options of being “agents of change” [34]. Lindemann, who also engages in feminist philosophy, highlights that bioethicists need to consider how “social pressures and personal idiosyncrasies affect moral judgments” [70] including one’s own biases and worldviews. She adds to this aspect the suggestion for bioethicists who do not engage in activism and activist bioethicists to cooperate and listen to each other.

With this review, we show more concrete ways for bioethicists to implement intersectionality in their research. As has already been mentioned, Moradi and Grzanka develop certain guidelines for “how to do intersectionality responsibly” [81] in the field of psychology. The authors intentionally use the term “guidelines”, borrowing from the American Psychological Association’s “guidelines for practice”, “to underscore that responsible stewardship of intersectionality requires active practice” [81]. The guidelines are organised alongside three major understandings of intersectionality, as has already been mentioned in the analysis: intersectionality (1) as a field of study (guidelines 1 and 2), (2) as an analytic strategy (guidelines 3–5), and (3) as a critical praxis for social justice (guidelines 6 and 7). The way they introduce these guidelines leaves room for adaptation and specification in bioethical research. One example could be guideline 2: “Make explicit the set of implicit values in knowledge production and critically evaluate how these values obscure intersectional analysis; expand the range of values and perspectives used to produce transformative knowledge and contribute to social change” [81]. Bioethicists could and should critically evaluate what kind of theories they use as standards in their work and research, where these theories originate from, in which contexts they are located, and which blind spots or implicit presumptions are included. The guideline demands “deep critical evaluation and re-envisioning of epistemology and to pursue interdisciplinary collaborations” [81].

Another interesting example is guideline 6: “Expand analytic approaches to intersectionality research and evaluate research for its level of community engagement and social impact throughout the research process, as opposed to only scholarly impact, generalizability, or statistical significance” [81]. Bioethicists could therefore ask themselves: Who is involved in the research process itself? Where are the research questions derived from and how can we have marginalised communities participate in the research? How do we define desired outcomes? In service for whom and why? These questions reflect the different functions of an intersectional perspective, can help to make bioethical research more participatory for marginalized groups to be heard, and ensure that research results benefit these communities. Thereby discrimination in the health-care sector can be analysed and stereotypes can be made visible and avoided in the future. An intersectional perspective therefore encourages bioethicists to learn from anti-racist, feminist, and queer critiques, enables them to jointly draw from these perspectives and address various power structures at the same time.

Finally, we mention some limitations of the study. One consideration worth mentioning is the meaning of the first author’s social location in society as a White, able-bodied cis-woman for the coding process and analysis. She recognised, for example, that the subcategory “Whiteness” was added comparably late to the system of categories, pointing towards her own blind spots and an impulse towards “othering” during the coding process. We argue that this form of self-reflection is part of an intersectional approach in qualitative research. The goal is not to appropriate the concept coming from Black feminism but to acknowledge its origin and stay self-critical during one’s own research processes.

Another aspect to reflect on is the fact that the articles included come from very different academic fields (bioethics and other journals). This might be a difficulty in terms of comparison but, at the same time, is a strength of this review. On the one hand, the articles include different areas and cultures of research and are written by authors from disciplines ranging from medicine and bioethics to sociology and gender studies, which might also be due to the interdisciplinarity of bioethical research itself. This means they use various methodological and theoretical approaches but also have differing understandings of scientific knowledge as such. On the other hand, the aim of this study, to give a first fruitful overview of the use of intersectionality for answering health-related (bio)-ethical questions was achieved. Moreover, the geographical scope of the research results included is very broad. While the majority of studies are based in a US-American and Canadian context, there are articles included from many other parts of the world, such as South Africa, Zimbabwe, Sri Lanka, and Brazil, as well as European countries, such as the UK, Switzerland, and Poland. An extension of the search to ethics monographs and textbooks would be possible. Subsequently, one could investigate thematic differences between journals and books. Our review has searched for ethics in dedicated ethics journals but also in journals from other fields, comprehensively covering the ethical literature in academic journals. An extension to books and book chapters was beyond the scope of this work. Lastly, because the use of the term “intersectional(ity)” was an inclusion criterion, it could be that research that is also intersectional in its analysis, for example, research on racism in bioethics which includes other dimensions, such as gender, could have been overlooked in this review.

## Conclusion

This SR provides a full overview of where and how the concept of intersectionality is used in bioethical research. It points out the breadth of academic fields and health-care disciplines working with the concept, from psychology and nursing, through gynaecology to palliative care and deaf studies. The use of the concept implies the consideration of the individual micro level, the institutional meso level, and the macro level of societal power structures. We developed the category system to provide insight into the wide scope of studies where intersectionality had already made its way into bioethical research. We identified several important functions that the concept of intersectionality fulfils in bioethical research, such as making inequities visible, resolving problems, improving health data collection, criticising, and self-reflecting. Intersectionality is also a critical praxis and, in this regard, close to the main aspects of bioethics, such as the (activist) goal of working towards justice in health care. Intersectionality aims to make research results relevant for respective communities and patients, it informs the development of policies and interventions, and situates analyses within their respective historical context.

However, an intersectional perspective is also suited to make bioethics theory and research itself more relevant as it strengthens the aspect of social justice, encourages self-reflection, and analyses that are more complex. It therefore creates meaningful research results that comprehensively address the realities of research subjects and enrich bioethical scientific discourse. As Kelly points out regarding the integration of feminist intersectionality and bioethics: “The goals of social action and justice on the one hand and identification of proximate causes and treatment of health problems on the other are not necessarily mutually exclusive” [61]. More interdisciplinary collaboration and community-based participatory research is needed as an area of common ground for an integrated approach. This systematic review shows how an intersectional perspective is useful in debates of these current controversial bioethical issues as well as for health-care providers themselves for working towards social justice, equal access, and less discrimination.

This review could be a basis for future research on how intersectionality could and should be used to answer health-related (bio)-ethical questions. It is a starting point for strengthening feminist perspectives in bioethics and fostering politically engaged bioethics. Concerning the question of how far bioethicists can and should be activists, we would like to underline some suggested strategies “such as courageously implementing policies and confronting the powerful” [89]. However, there is a need to discuss this in more detail using case studies and the sharing of experiences. Implementing an intersectional perspective in bioethical research means fostering a politicisation of bioethics, a willingness for social change and transformation, and arguing for new, more participatory forms of research and reflection on the former methods and traditional theories used. This includes critical self-reflection on the part of members of the field of bioethical research itself, the ways in which it is constituted, as well as which and how questions are asked and who answers them. Therefore, intersectionality is well suited to give innovative impulses for the field of bioethical research and theory itself.

In terms of future research, examples of best practices could help in discussing the concrete further implementation of intersectionality in certain health-care disciplines. Inspired by an article by Shields [93] from the field of psychology, there are two main goals for the field of bioethics in relation to intersectionality. First, feminist bioethics, as well as anti-racist, decolonial, and queer bioethics should be fostered and researchers using these perspectives should be brought together, promoted, and funded. Second, the intersectionality perspective should be implemented and brought into mainstream bioethics. Following this path, we see opportunities for broadening the perspective and enhancing the theoretical depth and methodological quality of bioethical scholarship.

### Electronic supplementary material

Below is the link to the electronic supplementary material.

Supplementary Material 1

## Data Availability

The datasets used and analysed during the current study are available from the corresponding author on reasonable request.

## Abbreviations

HIV/AIDS: Human Immunodeficiency Virus/Acquired Immunodeficiency Syndrome
LGBTQIA+: Lesbian, Gay, Bisexual, Trans, Queer, Inter und Asexual
PRISMA: Preferred Reporting Items for Systematic Reviews and Meta-Analyses
QCA: Qualitative Content Analysis
SR: Systematic Review
