# Supplementary material for: The concept of intersectionality in bioethics: a systematic review
Source: BMC Med Ethics. 2024 May 23;25:64. doi: 10.1186/s12910-024-01057-5 (PMC11112950; doi:10.1186/s12910-024-01057-5)
Supplement: Supplementary file 1 — Supplementary Material 1 [file 12910_2024_1057_MOESM1_ESM.docx]

**Supplements**

Supplement 1: System of main categories

| HOW | | | | | | Nr. of Articles |
| --- | --- | --- | --- | --- | --- | --- |
| **Function/Application purpose** | | | | | | **153** |
|  | Critical Praxis | | | | | 76 |
|  |  | | For justice, solidarity and representation | | | 40 |
|  |  | | Ensure results are relevant for respective communities | | | 16 |
|  |  | | To inform the development of policies and interventions | | | 19 |
|  |  | | For more complex and historicized analyses | | | 9 |
|  |  | | Foster politically engaged bioethics/pol. engagement | | | 7 |
|  | Critique & Self Reflection | | | | | 58 |
|  | To understand and explain a phenomenon | | | | | 76 |
|  | Ways of Applying | | | | | 30 |
|  |  | | Setting a research agenda | | | 13 |
|  |  | | Selecting tools and methods | | | 7 |
|  |  | | Analyzing & interpreting research data | | | 13 |
|  | As a framework to study health equity | | | | | 40 |
|  |  | | To prevent bias in research | | | 4 |
|  |  | | To provide a voice to marginalized/underprivileged people | | | 2 |
|  | To improve/develop something further | | | | | 46 |
|  | To describe a phenomenon/make something visible | | | | | 35 |
|  | As analytic strategy or disposition | | | | | 14 |
|  | For better health data collections | | | | | 14 |
|  | For interdisciplinary dialogue | | | | | 5 |
|  | To resolve a problem | | | | | 7 |
|  | For theorizing the relationship between research and activism | | | | | 7 |
|  | As “intersectionalities of influence” (on health & well-being) | | | | | 1 |
|  | As a field of study | | | | | 3 |
|  | As a form of ethics itself | | | | | 3 |
| **Social Dimensions** | | | | | | **185** |
|  | Sex, gender (identity), (cis-)sexism, misogyny, patriarchy | | | | | 165 |
|  |  | | Trans/transphobia, cisgenderism | | | 35 |
|  |  | | Gender beyond binary/non-binary | | | 12 |
|  |  | | Gender nonconforming (GNC) | | | 3 |
|  |  | | Gender expansive (GE) | | | 1 |
|  |  | | Intersex | | | 2 |
|  |  | | Gender performance | | | 2 |
|  | Race, ethnicity, racism, xenophobia | | | | | 169 |
|  |  | | Indigenous | | | 22 |
|  |  | | Whiteness | | | 25 |
|  |  | | Anti-asian hate/racism | | | 1 |
|  |  | | Islamophobia | | | 3 |
|  |  | | Ethnocentrism | | | 5 |
|  |  | | Colorism | | | 3 |
|  | Class/socio-economic status, classism/poverty | | | | | 144 |
|  |  | | Homelessness, housing conditions | | | 9 |
|  | Sexuality, sexual orientation, heteronormativity/heterosexism, homophobia | | | | | 109 |
|  |  | | LGBTQIA+/queer | | | 20 |
|  |  | | Men who have sex with men (MSM) | | | 4 |
|  | (Dis)ability, ableism | | | | | 87 |
|  |  | | Dwarfism | | | 1 |
|  |  | | Level of support needs | | | 2 |
|  |  | | Communication modes | | | 3 |
|  | Health status | | | | | 81 |
|  |  | | HIV-status | | | 17 |
|  |  | | Addiction e.g. drug use | | | 10 |
|  |  | | Victims of violence e.g. IPV, gender-based violence, reproductive coercion | | | 7 |
|  |  | | PTSD, trauma | | | 10 |
|  |  | | (un)insured | | | 5 |
|  |  | | Mentalism | | | 3 |
|  |  | | Neurodiverse | | | 1 |
|  |  | | Psychopathology | | | 1 |
|  | Culture | | | | | 70 |
|  | Age, generation, ageism | | | | | 87 |
|  | Geographical background and location, nativity | | | | | 67 |
|  | Historical dynamics (e.g. slavery, colonialism) | | | | | 70 |
|  | Migration, refugee status, citizenship, statelessness | | | | | 56 |
|  | Religion, spirituality, religious intolerance | | | | | 57 |
|  | Family status, parenthood, kinship | | | | | 45 |
|  | Language (skills) | | | | | 30 |
|  | Education/qualification | | | | | 42 |
|  | Body/anti-fatness | | | | | 22 |
|  | Nationality, nationalism | | | | | 24 |
|  | Occupation, (un)employment | | | | | 21 |
|  | Marital/relationship status | | | | | 14 |
|  | Professional status/occupation | | | | | 10 |
|  | Social capital/network, safety net | | | | | 6 |
|  | caste | | | | | 3 |
| **Challenges, Limitations, Critique of intersectionality** | | | | | | **56** |
|  | Methodological challenges | | | | | 32 |
|  | Theoretical challenges | | | | | 12 |
| WHERE | | | | | |  |
| **Levels** | | | | | | **171** |
|  | Individual level (micro) | | | | | 130 |
|  | Institutional level (meso) | | | | | 96 |
|  | Structural/societal level (macro) | | | | | 118 |
| **Health care disciplines and academic fields** | | | | | | **183** |
|  | Mental Health/Psychology | | | | | 74 |
|  |  | Psychotherapy and Counseling Psychology (Research) | | | | 24 |
|  |  |  | | | Marriage and Family Therapy (MFT) | 3 |
|  |  |  | | | “ethics of social justice in psychotherapy research” | 1 |
|  |  |  | | | (clinical) (multicultural) therapy supervision | 1 |
|  |  |  | | | Occupational therapy | 2 |
|  |  |  | | | Cross-racial therapy | 1 |
|  |  |  | | | Creative arts therapies | 4 |
|  |  | Psychology Research | | | | 13 |
|  |  | Trauma, PTSD | | | | 13 |
|  |  | Feminist & critical race work in Psychology | | | | 18 |
|  |  | Community Psychology (and Research) | | | | 4 |
|  |  | Child Mental Health | | | | 2 |
|  |  | Education and Training in Psychology | | | | 11 |
|  |  | Conversion | | | | 1 |
|  |  | Critical Disability Studies in Psychology | | | | 1 |
|  |  | “critical psychology” | | | | 4 |
|  |  | Personality psychology | | | | 1 |
|  |  | Autism | | | | 2 |
|  |  | Mental Health Nursing | | | | 1 |
|  |  | Addiction | | | | 5 |
|  |  | Psychoses, schizophrenia | | | | 1 |
|  |  | School Psychology | | | | 1 |
|  |  | Psychiatry | | | | 3 |
|  |  |  | | Child psychiatry & sexual exploitation | | 1 |
|  |  |  | | Geriatric psychiatry | | 1 |
|  | Medical-/Bioethics | | | | | 62 |
|  |  | Research Ethics | | | | 26 |
|  |  |  | | Representational ethics | | 2 |
|  |  |  | | Autism research ethics | | 1 |
|  |  |  | | Research compensation | | 1 |
|  |  | Care ethics | | | | 18 |
|  |  |  | | “intersectionality-inspired care ethics” | | 6 |
|  |  |  | | Feminist care ethics | | 4 |
|  |  | Rhetorics of Health and Medicine | | | | 1 |
|  |  | Principlism approach | | | | 7 |
|  |  | Medical education | | | | 1 |
|  |  | Virtue ethics | | | | 3 |
|  |  | Narrative ethics | | | | 3 |
|  |  | “bioethical antiracism” | | | | 2 |
|  | Nursing and Nursing Research | | | | | 21 |
|  |  | Nursing education | | | | 7 |
|  |  | Postcolonial, feminist perspectives in nursing research | | | | 7 |
|  |  | Health interventions research | | | | 2 |
|  |  | Research on female genital cutting | | | | 1 |
|  |  | Health Disparities Research | | | | 1 |
|  |  | Telephone nursing | | | | 1 |
|  |  | Maternity & Perinatal Care | | | | 1 |
|  | Gynecology, reproductive/sexual health | | | | | 27 |
|  |  | Assisted reproduction & surrogacy | | | | 3 |
|  |  | Reproductive justice & stratified reproduction | | | | 6 |
|  |  | Breastfeeding/Lactation Care | | | | 2 |
|  |  | Obstetric care and violence | | | | 1 |
|  |  | Birthing | | | | 2 |
|  |  | Abortion (and unintended pregnancies) | | | | 6 |
|  |  | Contraception | | | | 3 |
|  |  | Sterilization | | | | 2 |
|  |  | Menstruation | | | | 1 |
|  | HIV | | | | | 17 |
|  |  | HIV care | | | | 8 |
|  |  | HIV research(ers) | | | | 7 |
|  |  | Internalized HIV stigma | | | | 1 |
|  |  | HIV education | | | | 1 |
|  | Feminist perspectives | | | | | 52 |
|  | Public Health | | | | | 31 |
|  |  | Cancer screening | | | | 2 |
|  |  | Primary Health Care | | | | 3 |
|  |  | Health behavior (change) | | | | 1 |
|  |  | Healthcare Design Research | | | | 1 |
|  | Global/International Health | | | | | 9 |
|  |  | International migration of health workers/migrant health professionals | | | | 2 |
|  |  | Migrant health | | | | 1 |
|  |  | Global health epidemiology | | | | 1 |
|  |  | Medical geography | | | | 1 |
|  |  | Global Health Research | | | | 1 |
|  | Trans-focused research | | | | | 3 |
|  |  | Gender-Affirming Care (GAC) and Surgery (GAS) | | | | 2 |
|  | Disability studies | | | | | 15 |
|  |  | Autism studies | | | | 1 |
|  | De/post-colonial perspectives | | | | | 8 |
|  | Social work | | | | | 5 |
|  | Health services research | | | | | 7 |
|  |  | Patient engagement/public involvement | | | | 1 |
|  | COVID-related | | | | | 14 |
|  | (Social) Gerontology | | | | | 6 |
|  | Gendered violence (prevention) | | | | | 5 |
|  | Philosophy | | | | | 3 |
|  | Medical Humanities | | | | | 1 |
|  | Palliative Care | | | | | 2 |
|  |  | Medically assisted dying | | | | 1 |
|  | Health policy research | | | | | 2 |
|  | Genomic/Precision Medicine | | | | | 2 |
|  | Pediatrics | | | | | 3 |
|  | Gender Studies | | | | | 5 |
|  | Cardiovascular Medicine | | | | | 1 |
|  | Deaf studies | | | | | 1 |
|  | Medical sociology | | | | | 4 |
|  | Pharmacoeconomics | | | | | 1 |
|  |  | Cost effectiveness research | | | | 1 |
|  | Digital health and AI | | | | | 3 |
|  | Health professions education (research) | | | | | 1 |
|  | Criminology | | | | | 1 |

Supplement 2: Comprehensive list of all included articles (n=192)

| **x** | **Authors** | **Year** | **Title** | **Journal** |
| --- | --- | --- | --- | --- |
| 1 | Adler, J. M. | 2017 | Bringing the (disabled) body to personality psychology: A case study of Samantha | J. Pers. |
| 2 | Aguayo-Romero, R. A. | 2021 | (Re)centering Black Feminism Into Intersectionality Research | Am. J. Public Health |
| 3 | Alba, S. et al. | 2020 | Bridging research integrity and global health epidemiology (BRIDGE) guidelines: explanation and elaboration | BMJ Glob. Health |
| 4 | Albert, G. and Szilvasi, M. | 2017 | Intersectional Discrimination of Romani Women Forcibly Sterilized in the Former Czechoslovakia and Czech Republic | Health Hum. Rights J. |
| 5 | Al-Hamad, A. et al. | 2022 | The Potential of Merging Intersectionality and Critical Ethnography for Advancing Refugee Women's Health Research. ANS Adv Nurs Sci 45(2): 143-154. | ANS Adv. Nurs. Sci. |
| 6 | Ambrogi, I. et al. | 2022 | Reflections on research ethics in a public health emergency: Experiences of Brazilian women affected by Zika." | Dev. World Bioeth. |
| 7 | Ammann, C. et al. | 2020 | Negotiating social differences and power geometries among healthcare professionals in a Swiss hospital | Gend. Place Cult. |
| 8 | Antequera, A. et al. | 2021 | Improving Social Justice in COVID-19 Health Research: Interim Guidelines for Reporting Health Equity in Observational Studies. | Int. J. Environ. Res. Public Health |
| 9 | Asakura, K. and Maurer, K. | 2018 | Attending to Social Justice in Clinical Social Work: Supervision as a Pedagogical Space | Clin. Soc. Work J. |
| 10 | Attrash-Najjar, A. and Katz, C. | 2022 | Child Sexual Abuse Studies in Arab Societies: A Systematic Review and Directions for Future Research. | Trauma Violence Abuse |
| 11 | Aya Pastrana, N. et al. | 2020 | The gender responsiveness of social marketing interventions focused on neglected tropical diseases | Glob. Health Action |
| 12 | Bannink Mbazzi, F. and Kawesa, E. S. | 2022 | 'Impairments of the brain': Global South perspectives on childhood neurodevelopmental disability | Dev. Med. Child Neurol. |
| 13 | Barned, C. et al. | 2019 | Addressing the Practical Implications of Intersectionality in Clinical Medicine: Ethical, Embodied and Institutional Dimensions | Am. J. Bioeth. |
| 14 | Bartos, A. E. | 2019 | Introduction: Stretching the boundaries of care | Gend. Place Cult. |
| 15 | Berghs, M. et al. | 2019 | Rights to social determinants of flourishing? A paradigm for disability and public health research and policy | BMC Public Health |
| 16 | Berkhout, S. G. and Richardson, L. | 2020 | Identity, politics, and the pandemic: Why is COVID a disaster for feminism(s)? | Hist. Philos. Life Sci. |
| 17 | Bhakuni, H. | 2021 | Reproductive justice: Non-interference or non-domination? | Dev. World Bioeth. |
| 18 | Borras, A. M. | 2021 | Toward an Intersectional Approach to Health Justice | Int. J. Health Serv. |
| 19 | Bounds, D. T. et al. | 2020 | Adapting a family intervention to reduce factors for sexual exploitation | Child Adolesc. Psychiatry Ment. Health |
| 20 | Bowleg, L. | 2012 | The Problem With the Phrase Women and Minorities: Intersectionality - an Important Theoretical Framework for Public Health | Am. J. Public Health |
| 21 | Bowleg, L. | 2021 | The Master's Tools Will Never Dismantle the Master's House: Ten Critical Lessons for Black and Other Health Equity Researchers of Color." | Health Educ. Behav. |
| 22 | Brandão, E. R. and Cabral C. S. | 2021 | Youth, gender and reproductive justice: health inequities in family planning in Brazil's Unified Health System. | Ciência & Saúde coletiva Journal |
| 23 | Breakey, H. et al. | 2021 | Migrant health professionals' systemic human rights vulnerabilities | Int. Migr. |
| 24 | Brewster, M. E. and López Molina, D. A. | 2021 | Centering Matrices of Domination: Steps Toward a More Intersectional Vocational Psychology | J. Career Assess. |
| 25 | Brünig, L. and Salloch, S. | 2022 | Making Structural Discrimination Visible: A Call for Intersectional Bioethics. | Am. J. Bioeth. |
| 26 | Bryant-Davis, T. | 2019 | The Cultural Context of Trauma Recovery: Considering Posttraumatic Stress Disorder Practice Guideline and Intersectionality | Psychotherapy |
| 27 | Burger, K. et al. | 2022 | Reproductive justice and black lives: A concept analysis for Public Health Nurs. | Public Health Nurs. |
| 28 | Burrow, S. et al. | 2018 | Vulnerability, Harm, and Compromised Ethics Revealed by the Experiences of Queer Birthing Women in Rural Healthcare | J. Bioeth. Inq. |
| 29 | Cascio, M.A. et al. | 2021 | Making Autism Research Inclusive by Attending to Intersectionality: a Review of the Research Ethics Literature | Rev. J. Autism Dev. Disord. |
| 30 | Chandrika Millner, U. | 2015 | Revitalizing Communication Between Split Identities and Psychotic Processes in a South-Asian Treatment Dyad | Women Ther. |
| 31 | Cheema, A. W. et al. | 2019 | Multiple Marginalizations: What Bioethics Can Learn From Black Feminism | Am. J. Bioeth. |
| 32 | Choby, A. A. and Clark, A. M. | 2014 | Improving health: Structure and agency in health interventions | Nurs. Philos. |
| 33 | Clark, N. and Saleh, N. | 2019 | Applying Critical Race Feminism and Intersectionality to Narrative Inquiry | ANS Adv. Nurs. Sci. |
| 34 | Cole, E. R. | 2009 | Intersectionality and Research in Psychology | Am. Psychol. |
| 35 | Collins, A. B. et al. | 2017 | “We’re giving you something so we get something in return”: perspectives on research participation and compensation among people living with HIV who use drugs | Int. J. Drug Policy |
| 36 | Crocker, S. | 2021 | A Call for Intersectional Healthcare Design Research | HERD |
| 37 | Cuesta, M. and Rämgård, M. | 2016 | Intersectional perspective in elderly care | Int. J. Qual. Stud. Health. Well-being |
| 38 | Cuijpers, F. et al. | 2022 | ""Who Am I to Judge These Things": Intersectional Dimensions of Self-Silencing of People with a Neuromuscular Disease in a Clinical Trial." | Int. J. Fem. Approaches Bioeth. |
| 39 | Currier, J. M. et al. | 2022 | Enhancing competencies for the ethical integration of religion and spirituality in Psychol. Serv. | Psychol. Serv. |
| 40 | Davidson, M. M. and Hauser, C. T. | 2015 | Multicultural Counselling Meets Potentially Harmful Therapy: The Complexity of Bridging Two Discourses | Couns. Psychol. |
| 41 | Davy, Z. | 2011 | The promise of intersectionality theory in primary care | Qual. Prim. Care |
| 42 | De Hertogh, L. B. | 2018 | Feminist Digital Research Methodology for Rhetoricians of Health and Medicine | J. Bus. Tech. Commun. |
| 43 | De Sousa, I. and Varcoe, C. | 2022 | Centering Black feminist thought in nursing praxis | Nurs. Inq. |
| 44 | Dee, E. C. et al. | 2021 | Anti-Asian American Racism: A Wake-Up Call for Population-Based Cancer Research | Cancer Epidemiology, Biomarkers & Prevention |
| 45 | Delgado, J. et al. | 2022 | Bias in algorithms of AI systems developed for COVID-19: A scoping review | J. Bioeth. Inq. |
| 46 | Dent, J. R. | 2006 | Motherhood vs. Patienthood: a conflict of identities | J. Med. Ethics |
| 47 | Dineen, K. K. and Pendo, E. | 2022 | Engaging Disability Rights Law to Address the Distinct Harms at the Intersection of Race and Disability for People with Substance Use Disorder. | J. Law Med. Ethics |
| 48 | Dobson, K. S. | 2022 | Diversity and Canadian Psychology: An Evolving Relationship | Can. Psychol. |
| 49 | Dubé, K. et al. | 2022 | Considerations for Increasing Racial, Ethnic, Gender, and Sexual Diversity in HIV Cure-Related Research with Analytical Treatment Interruptions: A Qualitative Inquiry." | AIDS Res. Hum. Retroviruses |
| 50 | Dutta, U. | 2016 | Prioritzing the Local in an Era of Globalization: A Proposal for Decentering Community Psychology | Am. J. Community Psychol. |
| 51 | Eagle, G. and Long, C. | 2011 | In our culture, in our gender: Implications of the culture/gender interface for South African psychotherapists | Fem. Psychol. |
| 52 | Eilenberger, H.-G. et al. | 2019 | Age Difference in the Clinical Encounter: Intersectionality and Phenomenology | Am. J. Bioeth. |
| 53 | Engelman, A. et al. | 2019 | State of the Profession: The Landscape of Disability Justice, Health Inequities, and Access for Patients with Disabilities | ANS Adv. Nurs. Sci. |
| 54 | Estrella, K. | 2022 | Commentary: Troubling care. | Arts Psychother. |
| 55 | Ettorre, E. | 2018 | Women-only treatment? Epistemologies of ignorance, intersectionality and the need for a feminist embodiment approach | Addiction |
| 56 | Figueroa, C. A. et al. | 2021 | The need for feminist intersectionality in digital health. | Lancet Digit. Health |
| 57 | Fine, M. | 2019 | Critical Disability Studies: Looking Back and Forward | J. Soc. Issues |
| 58 | Fine, M. and Torre, M.E. | 2019 | Critical Participatory Action Research: A Feminist Project for Validity and Solidarity | Psychol. Women Q. |
| 59 | Fine, M. et al. | 2021 | Critical participatory action research: Methods and praxis for intersectional knowledge production." | J. Couns. Psychol. |
| 60 | Flaherty, A. J. et al. | 2020 | Should Gender-Affirming Surgery be Prioritized During the Covid-19 Pandemic? | Otolargyngol. Head Neck Surg. |
| 61 | Flynn, A. W. P. et al. | 2021 | When the political is professional: Civil disobedience in psychology. | Am. Psychol. |
| 62 | Frazier, K. E. | 2012 | Reclaiming the Person: Intersectionality and Dynamic Social Categories Through a Psychological Lens | Integr. Psychol. Behav. Sci. |
| 63 | Freeman, R. et al. | 2017 | Critical race theory as a tool for understanding poor engagement along the HIV care continuum among African American/Black and Hispanic persons living with HIV in the United States: a qualitative exploration | Int. J. Equity Health |
| 64 | Gaard, G. | 2022 | Queering Environmental Justice Through an Intersectional Lens. | Gend. Place Cult. |
| 65 | Gerlach, A. J. | 2015 | Sharpening our critical edge: Occupational therapy in the context of marginalized populations | Can. J. Occup. Ther. |
| 66 | Gesink, D. et al. | 2014 | Who are the under- und never-screened for cancer in Ontario:a qualitative investigation | BMC Public Health |
| 67 | Gilmore-Bykovskyi, A. et al. | 2022 | Traversing the Aging Research and Health Equity Divide: Toward Intersectional Frameworks of Research Justice and Participation. | Gerontologist |
| 68 | Gopalakrishna, M. | 2022 | Practicing in an expanded paradigm: Case examples and ethical anchors for creative arts therapists working in community-based social justice contexts. | Arts Psychother. |
| 69 | Grachev, K. et al. | 2022 | Feminist contributions on sexual experiences of women with serious mental illness: a literature review. | Arch. Womens Ment. Health |
| 70 | Grigorivich, A. | 2016 | The meaning of quality care in home-care settings: older lesbian and bisexual women´s perspectives | Scand. J. Caring Sci. |
| 71 | Griswold, M. K. and Pagano-Therrien, J. | 2020 | Women living with HIV in High-Income Countries and the Deeper Meaning of Breastfeeding Avoidance: A Metasynthesis | J. Hum. Lact. |
| 72 | Grzanka, P. R. and Brian, J. D. | 2019 | Clinical Encounters: The Social Justice Question in Intersectional Medicine | Am. J. Bioeth. |
| 73 | Grzanka, P. R. and Cole, E. R. | 2021 | An Argument for Bad Psychology: Disciplinary Disruption, Public Engagement, and Social Transformation." | Am. Psychol. |
| 74 | Grzanka, P. R. et al. | 2016 | My Bioethics Will Be Intersectional or It Will Be (Bleep) | Am. J. Bioeth. |
| 75 | Grzanka, P. R. et al. | 2020 | "Sincerely Held Principles" or Prejudice? The Tennessee Counseling Discrimination Law | Couns. Psychol. |
| 76 | Grzanka, P. R. et al. | 2019 | Conscience Clauses and Sexual and Gender Minority Health Care: A Case Study | J. Couns. Psychol. |
| 77 | Grzanka, P. R. et al. | 2017 | Intersectionality Research in Counseling Psychology | J. Couns. Psychol. |
| 78 | Gunnarson Payne, J. | 2018 | Autonomy in altruistic surrogacy, conflicting kinship grammars and intentional multilinear kinship | Reprod. Biomed. Soc. |
| 79 | Guruge, S. and Khanlou, N. | 2004 | Intersectionalities of influence: Researching the health of immigrant and refugee women | Can. J. Nurs. Res. |
| 80 | Gutierrez, D. | 2018 | The Role of Intersectionality in Marriage and Family Therapy Multicultural Supervision | Am. J. Fam. Ther. |
| 81 | Haarlammert, M. et al. | 2017 | Inside Out: Representational Ethics and Diverse Communities | Am. J. Community Psychol. |
| 82 | Hall, J. M. and Carlson, K. | 2016 | Marginalization - A Revisitation with Integration of Scholarship on Globalization, Intersectionality, Privilege, Microagressions, and Implicit Biases | ANS Adv. Nurs. Sci. |
| 83 | Hankivsky, O. | 2014 | Rethinking Care Ethics: On the Promise and Potential of an Intersectional Analysis | Am. Polit. Sci. Rev. |
| 84 | Henderson, D. | 1997 | Intersecting Race and Gender in Feminist Theories of Women's Psychological Development | Issues in Mental Health Nursing |
| 85 | Henrickson, M. et al. | 2020 | Research Ethics with Gender and Sexually Diverse Persons | Int. J. Environ. Res. Public Health |
| 86 | Hipp, T. N. et al. | 2019 | From Conversion Towards Affirmation: Psychology, Civil Rights, and Experiences of Gender-Diverse Communities in Memphis | Am. Psychol. |
| 87 | Höglund, A. T. et al. | 2018 | From denial to awareness: a conceptual model for obtaining equity in healthcare | Int. J. Equity Health |
| 88 | Ibánez-Carrasco, F. et al. | 2020 | Universities without Walls: A Blended Delivery Approach to Training the Next Generation of HIV Researchers in Canada | Int. J. Environ. Res. Public Health |
| 89 | Ion, R. et al. | 2018 | Teaching ethics: Intersectionality, care failure and moral courage | Nurse Educ. Today |
| 90 | Kanagasingam, D. et al. | 2022 | 'It's not just to treat everybody the same': A social justice framework for caring for larger patients in healthcare practice. | Soc. Health Illn. |
| 91 | Kapiriri, L. and Razavi, S. D. | 2022 | Equity, justice, and social values in priority setting: a qualitative study of resource allocation criteria for global donor organizations working in low-income countries. | Int. J. Equity Health |
| 92 | Kelly, U. A. | 2009 | Integrating Intersectionality and Biomedicine in Health Disparities Research | ANS Adv. Nurs. Sci. |
| 93 | Kennedy, C. E. et al. | 2013 | "They are human beings, they are Swazi": intersecting stigmas and the positive health, dignity and prevention needs of HIV-positve men who have sex with men in Swaziland | J. Int. AIDS Soc. |
| 94 | Khader, S. J. | 2013 | Intersectionality and the Ethics and transnational commercial surrogacy | Int. J. Fem. Approaches Bioeth. |
| 95 | Koch, A. and Kozhumam, A. | 2022 | Adultification of Black children negatively impacts their health: Recommendations for health care providers." | Nurs. Forum |
| 96 | Kuri, E. And Schormans, A. F. | 2022 | Pivotal care practices: Care ethics in inclusive arts-based research with people labelled/with intellectual disability during the COVID-19 pandemic | Arts Psychother. |
| 97 | Lamprell, G. and Braithwaite, J. | 2017 | Mainstreaming gender and promoting intersectionality in Papua New Guinea's health policy: a triangulated analysis applying data-mining and content analytic techniques | Int. J. Equity Health |
| 98 | Lanphier, E. and Anani, U. | 2019 | Narrative Ethics and Intersectionality | Am. J. Bioeth. |
| 99 | Lazaridou, F. and Fernando, S. | 2022 | Deconstructing institutional racism and the social construction of whiteness: A strategy for professional competence training in culture and migration mental health." | Transcult. Psychiatry |
| 100 | Linton, J. M. et al. | 2019 | Pediatricians Awakened: Addressing Family Immigration Status as a Critical and Intersectional Social Determinant of Health | Am. J. Bioeth. |
| 101 | Lokugamage, A. U. | 2020 | Decolonising ideas of healing in medical education | J. Med. Ethics |
| 102 | Lopez, P. J. | 2019 | Toward a care ethical approach to access to health care in neoliberal times | Gend. Place Cult. |
| 103 | Macer, D. R. J. | 2019 | Is there Cross-cultural Evidence for an Association between Intersectionality and Bioethical Decision Making? Not Yet, but Awaiting Advances in Mental Mapping | Am. J. Bioeth. |
| 104 | Macleod, C. I. | 2019 | Expanding Reproductive Justice through a supportability reparative justice framework: the case of abortion in South Africa | Cult. Health Sex. |
| 105 | Martino, E. et al. | 2020 | Planning with care: Violence prevention policy at the intersection of invisibilities | Cities |
| 106 | Maykut, C. | 2021 | Deconstructing identity: Professional relationships for sustaining morally habitable workplaces | Arch. Psychiatr. Nurs. |
| 107 | Mbazzi, F. B. and Kawesa, E. S. | 2022 | 'Impairments of the brain': Global South perspectives on childhood neurodevelopmental disability. | Dev. Med. Child Neurol. |
| 108 | McCabe, K. | 2022 | Criminalization of Care: Drug Testing Pregnant Patients. | J. Health Soc. Behav. |
| 109 | McPherson, C. M. and McGibbon, E. A. | 2010 | Addressing the Determinants of Child Mental Health: Intersectionality as a Guide to Primary Health Care Renewal | Can. J. Nurs. Res. |
| 110 | McRae, J. and Onukwugha, E. | 2021 | Why the Gap in Evaluating the Social Constructs and the Value of Medicines?" | Pharmacoeconomics |
| 111 | Merz, S. et al. | 2021 | Intersectionality and eco-social theory: a review of potentials for public health knowledge and social justice. | Crit. Public Health |
| 112 | Mfoafo-M'Carthy, M. and Grischow, J. | 2022 | Hierarchy and inequality in research: Navigating the challenges of research in Ghana. | Qual. Res. |
| 113 | Midoun, M. et al. | 2016 | How intersectional constructions of sexuality, culture, and masculinity shape identities and sexual decision-making among men who have sex with men in coastal Kenya | Cult. Health Sex. |
| 114 | Migala, S. and Flick, U. | 2020 | Altern und Sterben in Diversität - Implikationen einer intersektionalen Perspektive für die Analyse pflegepolitischer Diskurse | Z. Gerontol. Geriatr. |
| 115 | Mitchell, W. et al. | 2021 | The Human Right to Justice for Older Persons With Mental Health Conditions | Am. J. Geriatr. Psychiatry |
| 116 | Monaghan, L. F. and Malson, H. | 2013 | It's worse for women and girls': negotiating embodied masculinities through weight-related talk | Crit. Public Health |
| 117 | Moore, I. et al. | 2022 | The intersection of autism and gender in the negotiation of identity: A systematic review and metasynthesis | Fem. Psychol. |
| 118 | Moradi, B. and Grzanka, P. R. | 2017 | Using intersectionality responsibly: Toward critical epistemology, structural analysis, and social justice activism | J. Couns. Psychol. |
| 119 | Morris, D. J. et al. | 2022 | Moral injury in secure mental healthcare part II: experiences of potentially morally injurious events and their relationship to wellbeing in health professionals in secure services. | J. Forens. Psychiatry Psychol. |
| 120 | Mukherjee, T. I. et al. | 2021 | Reproductive justice in the time of COVID-19: a systematic review of the indirect impacts of COVID-19 on sexual and reproductive health." | Reprod. Health |
| 121 | Muntaner, C. and Augustinavicius, J. | 2019 | Intersectionality: A Scientific Realist Critique | Am. J. Bioeth. |
| 122 | Nelson, C. A. | 2006 | Of Eggshells and Thin-Skulls: A consideration of racism-related mental illness impacting Black women | Int. J. Law Psychiatry |
| 123 | Niemann, Y. F. | 2020 | The Obfuscation of the Realities of Women of Color Due the False Dichotomy Phrasing of "Women and Minorities" | Women Ther. |
| 124 | Nungsari, M. et al. | 2021 | Understanding the impact of the COVID-19 outbreak on vulnerable populations in Malaysia through an ethical lens: A study of non-state actors involved in aid distribution. | Wellcome Open Res. |
| 125 | Nyatsanza, T. and Wood, L. | 2017 | Problematizing Official Narratives of HIV and AIDS education in Scotland and Zimbabwe | SAHARA-J |
| 126 | Oswald, A. G. et al. | 2021 | Intersectional expansiveness borne at the neuroqueer nexus. | Psychol. Sex. |
| 127 | Pantelic, M. et al. | 2019 | It's not "all in your head": critical knowledge gaps on internalized HIV stigma and a call for integrating social and structural conceptualizations | BMC Infect. Dis. |
| 128 | Paquin, J. D. et al. | 2019 | Toward a Psychotherapy Science for All: Conducting Ethical and Socially Just Research | Psychotherapy |
| 129 | Parikh, S. A. | 2012 | They Arrested me for Loving a Schoolgirl: Ethnography, HIV and a feminist Assessment of the Age of Consent Law as a Gender-based Structural Intervention in Uganda | Soc. Sci. Med. |
| 130 | Paynter, M. J. et al. | 2018 | A critical review of human milk sharing using an intersectional feminist framework: Implications for practice | J. Clin. Nurs. |
| 131 | Perera, D. et al. | 2018 | "When helpers hurt": women's and midwives' stories of obstetric violence in state health institutions, Colombo district, Sri Lanka | BMC Pregnancy Childbirth |
| 132 | Plamondon, K. M. and Bisung, E. | 2019 | The CCGHR Principles for Global Health Research: Centering equity in research, knowledge translation, and practice | Soc. Sci. Med. |
| 133 | Pooley, E. A. and Beagan, B. L. | 2021 | The Concept of Oppression and Occupational Therapy: A Critical Interpretive Synthesis. | Can. J. Occup. Ther. |
| 134 | Price, K. | 2011 | It's Not Just About Abortion: Incorporating Intersectionality in Research About Women of Color and Reproduction | Women's Health Issues |
| 135 | Pritchard, E. | 2019 | Female Researcher Safety: the difficulty of recruiting participants at conventions for people with dwarfism | Int. J. Soc. Res. Methodol. |
| 136 | Raghuram, P. | 2019 | Face and feminist care ethics: intersectionality as a method | Gend. Place Cult. |
| 137 | Rasmus, S. M. et al. | 2020 | An intervention science to advance underrepresented perspectives and indigenous self-determination in health | Prev. Sci. |
| 138 | Ratnapalan, S. and Haldane, V. | 2022 | We go farther together: practical steps towards conducting a collaborative autoethnographic study | JBI Evid. Implement. |
| 139 | Ray, K. S. | 2019 | Intersectionality and Power Imbalances Clinicians of Color Face When Patients Request White Clinicians | Am. J. Bioeth. |
| 140 | Reimer-Kirkham, S. | 2014 | Nursing Research on Religion and Spirituality Through a Social Justice Lens | ANS Adv. Nurs. Sci. |
| 141 | Ren, J. and Feagin, J. | 2021 | Face mask symbolism in anti-Asian hate crimes | Ethn. Racial Stud. |
| 142 | Reynolds, J. M. | 2020 | Health for Whom? Bioethics and the Challenge of Justice for Genomic Medicine | Hastings Cent. Rep. |
| 143 | Richter, A.S. and Kricheldorff, C. | 2020 | Alter(n) und Intersektionalität | Z. Gerontol. Geriatr. |
| 144 | Rogers, J. and Kelly, U.A. | 2011 | Feminist intersectionality: Bringing social justice to health disparities research | Nurs. Ethics |
| 145 | Rosenthal, L. | 2016 | Incorporating Intersectionality into Psychology: An Opportunity to Promote Social Justice and Equity | Am. Psychol. |
| 146 | Rotz, S. et al. | 2022 | Toward intersectional and culturally relevant sex and gender analysis in health research | Soc. Sci. Med. |
| 147 | Rueda, J. | 2021 | Ageism in the COVID-19 pandemic: age-based discrimination in triage decisions and beyond. | Hist. Philos. Life Sci. |
| 148 | Ruth, R. | 2017 | Ethics, Risk Managament and an Intersectional View of LGBT Mental Health | Psychiatry |
| 149 | Saleh, N. et al. | 2022 | Using Narrative Inquiry to Understand Anti-Muslim Racism in Canadian Nursing | Can. J. Nurs. Res. |
| 150 | Seedall, R. B. et al. | 2014 | Diversity, Social Justice and Intersectionality Trends in C/MFT: A Content Analysis of three Family Therapy Journals, 2004-2011 | J. Marital Fam. Ther. |
| 151 | Seelman, K. L. et al. | 2021 | "Predictors of healthcare mistreatment among transgender and gender diverse individuals: Are there different patterns by patient race and ethnicity? | Soc. Work Health Care |
| 152 | Seyyed-Kalantari, L. et al. | 2021 | Underdiagnosis bias of artificial intelligence algorithms applied to chest radiographs in under-served patient populations. | Nat. Med. |
| 153 | Shelton, S. A. and Lester, A. O. S. | 2020 | A narrative exploration of the importance of intersectionality in a Black trans woman's mental health experiences | Int. J. Transgend. Health |
| 154 | Shimmin, C. | 2017 | Moving towards a more inclusive patient and public involvement in health research paradigm: the incorporation of trauma-informed intersectional analysis | BMC Health Sev. Res. |
| 155 | Shin, R. Q. et al. | 2017 | The Intersectionality Framework and Identity Intersections in the J. Couns. Psychol. and Couns. Psychol.: A Content Analysis | J. Couns. Psychol. |
| 156 | Sikka, T. | 2021 | Barriers to Access: A Feminist Analysis of Medically Assisted Dying and the Experience of Marginalized Groups | Omega J. Death Dying |
| 157 | Singh, P. et al. | 2014 | African Kaposi's Sarcoma in the Light of Global AIDS: Antiblackness and Viral Visibility | J. Bioeth. Inq. |
| 158 | Sontan, O. | 2021 | Care Ethics versus the CARES Act. | Hastings Cent. Rep. |
| 159 | Squier, S. M. | 2007 | Beyond Nescience: the intersectional insights of health humanities | Perspect. Biol. Med. |
| 160 | St. John, M. S. | 2019 | Reconceiving the field: Infant mental health, intersectionality, and reproductive justice | Infant Mental Health J. |
| 161 | Sullivan, A. L. et al. | 2021 | A call to action for school psychology to address COVID-19 health disparities and advance social justice. | J. Sch. Psychol. |
| 162 | Suzuki, L. A. et al. | 2019 | Counseling Psychology and the Amelioration of Oppression: Translating our Knowledge into Action | Couns. Psychol. |
| 163 | Switzer, S. et al. | 2021 | Picturing Participation: Catalyzing Conversations About Community Engagement in HIV Community-Based Organizations | Health Educ. Behav. |
| 164 | Syed, M. | 2010 | Disciplinarity and Methodology in Intersectionality Theory and Research | Am. Psychol. |
| 165 | Talwar, S. and Sajnani, N. | 2022 | Intersectionality and the Ethics of Care in the creative arts therapies. | Arts Psychother. |
| 166 | Tam, M. W. | 2021 | Queering reproductive access: reproductive justice in assisted reproductive technologies | Reprod. Health |
| 167 | Taylor, L. A. et al. | 2021 | Should a Healthcare System Facilitate Racially Concordant Care for Black Patients? | Pediatrics |
| 168 | Tseng, V. and Lee, R. M. | 2021 | From margin to center: An Asian Americanist psychology. | Am. Psychol. |
| 169 | van den Berg, Z. D. and Allen, P.B. | 2022 | Specters of whiteness: Radical care for ghostly matters in art therapy | Arts Psychother. |
| 170 | van Herk, K. A. et al. | 2011 | Examining our privileges and oppressions: incorporating an intersectionality paradigm into nursing | Nurs. Inq. |
| 171 | Vincent, B. W. | 2018 | Studying trans: recommendations for ethical recruitment and collaboration with transgender participants in academic research | Psychol. Sex. |
| 172 | Vissandjée, B. et al. | 2017 | Health and legal literacy for migrants: twinned strands woven in the cloth of social justice and the human right to health care | BMC Int. Health Hum. Rights |
| 173 | Voith, L. A. et al. | 2020 | Using a Trauma-Informed, Socially Just Research Framework with Marginalized Populations: Practices and Barriers to Implementation | Soc. Work Res. |
| 174 | Watson, K. S. et al. | 2022 | Adapting a conceptual framework to engage diverse stakeholders in genomic/precision medicine research | Health Expect. |
| 175 | Watts-Jones, T. D. | 2010 | Location of Self: Opening the Door to Dialogue on Intersectionality in the Therapy Process | Fam. Process |
| 176 | Weitzel, J. et al. | 2020 | The Role of Nurses as Allies against Racism and Discrimination | ANS Adv. Nurs. Sci. |
| 177 | Werunga, J. et al. | 2016 | A Decolonizing Methodology for Health Research on Female Gential Cutting | ANS Adv. Nurs. Sci. |
| 178 | Wesp, L. M. et al. | 2018 | An Emancipatory Approach to Cultural Competency | ANS Adv. Nurs. Sci. |
| 179 | Weßel, M. | 2022 | Feminist approach to geriatric care: comprehensive geriatric assessment, diversity and intersectionality. | Med. Health Care Philos. |
| 180 | White, J. et al. | 2021 | The Integration of Sex and Gender Considerations Into Biomedical Research: Lessons From International Funding Agencies. | J. Clin. Endocrinol. Metab. |
| 181 | Whittal, A. and Böckmann, M. | 2018 | Internationale Rekrutierung und Migration von Ärztinnen, Ärzten und Personal in Gesundheitsfachberufen: ein qualitatives Scoping Review der Public Health Literatur | Ethik Med. |
| 182 | Williams, F. | 2018 | Care: Intersections of scales, inequalities and crises | Curr. Sociol. |
| 183 | Wilmberly, J. M. | 2019 | Virtue ethics and the commitment to learn: overcoming disparities faced by transgender individuals | Philos. Ethics, Humanit. Med. |
| 184 | Wilson, Y. et al. | 2019 | Intersectionality in Clinical Medicine: The Need for a Conceptual Framework | Am. J. Bioeth. |
| 185 | Wilson, Y. et al. | 2019 | Broadening the conversation about intersectionality in Clinical Medicine | Am. J. Bioeth. |
| 186 | Wolowicz, A. et al. | 2020 | Women with disabilities and access to gynaecological services in Poland | Disabil. Soc. |
| 187 | Wright, T. and Wright, K. | 2022 | Proposing a justice approach to ethics of care in art psychotherapy. | Arts Psychother. |
| 188 | Wu, J. et al. | 2019 | Looking back while moving forward: a justice-based, intersectional approach to research on contraception and disability | Contraception |
| 189 | Wyatt, J. P. and Ampadu, G.G. | 2022 | Reclaiming Self-care: Self-care as a Social Justice Tool for Black Wellness | Community Ment. Health J. |
| 190 | Wyatt, T. R. et al. | 2022 | Intersectionality: a means for centering power and oppression in research | Adv. Health Sci. Educ. |
| 191 | Yip, S. H. et al. | 2022 | Emergency assistance in situations of abuse, neglect, and self-neglect: exploring the complexity and challenges." | J. Elder Abuse Negl. |
| 192 | Zubair, M. and Norris, M. | 2015 | Perspectives on ageing, later life and ethnicity: ageing research in ethnic minority contexts | Ageing Soc. |
